# Breeding Strategy Shapes the Composition of Bacterial Communities in Female Nile Tilapia Reared in a Recirculating Aquaculture System

**DOI:** 10.3389/fmicb.2021.709611

**Published:** 2021-09-10

**Authors:** Yousri Abdelhafiz, Jorge M. O. Fernandes, Simone Larger, Davide Albanese, Claudio Donati, Omid Jafari, Artem V. Nedoluzhko, Viswanath Kiron

**Affiliations:** ^1^Faculty of Biosciences and Aquaculture, Nord University, Bodø, Norway; ^2^Unit of Computational Biology, Fondazione Edmund Mach, San Michele all’Adige, Italy; ^3^International Sturgeon Research Institute, Iranian Fisheries Science Research Institute, Agricultural Research, Education and Extension Organization, Rasht, Iran

**Keywords:** breeding, Nile tilapia, microbiome, 16S amplicon, whole-genome sequencing, core microbiome

## Abstract

In industrial animal production, breeding strategies are essential to produce offspring of better quality and vitality. It is also known that host microbiome has a bearing on its health. Here, we report for the first time the influence of crossbreeding strategy, inbreeding or outbreeding, on the buccal and intestinal bacterial communities in female Nile tilapia (*Oreochromis niloticus*). Crossbreeding was performed within a family and between different fish families to obtain the inbred and outbred study groups, respectively. The genetic relationship and structure analysis revealed significant genetic differentiation between the inbred and outbred groups. We also employed a 16S rRNA gene sequencing technique to understand the significant differences between the diversities of the bacterial communities of the inbred and outbred groups. The core microbiota composition in the mouth and the intestine was not affected by the crossbreeding strategy but their abundance varied between the two groups. Furthermore, opportunistic bacteria were abundant in the buccal cavity and intestine of the outbred group, whereas beneficial bacteria were abundant in the intestine of the inbred group. The present study indicates that crossbreeding can influence the abundance of beneficial bacteria, core microbiome and the inter-individual variation in the microbiome.

## Introduction

Animals are bred for food, fibers, transport, protection, company as well as for other purposes such as scientific research (Flint and Woolliams, 2008). Domestication of different animals, mainly livestock species started several years ago and presently crossbreeding programs are essential tools to improve the productivity, efficiency, and sustainability of domesticated animals (Hill, 2014, 2016). Initially, livestock were selected based on desired phenotypic traits. Over the past 50years, there has been a remarkable increase in livestock production due to the improvement in breeding practices and better understanding of genetics. Genetics plays an important role in modern breeding programs, which combine basic breeding concepts and emerging technologies (Schultz et al., 2020).

Crossbreeding of farmed animals and agricultural plants is well-established compared to those of farmed aquatic animals (D’ambrosio et al., 2019; Gratacap et al., 2019). However, the production of fish based on crossbreeding programs is expected to increase as the farming of fish such as Nile tilapia (*Oreochromis niloticus*) and Atlantic salmon (*Salmo salar*) is expanding rapidly (Gjedrem et al., 2012; Gjedrem and Rye, 2018; Mehar et al., 2019). Several strategies such as selective breeding have been implemented to increase the production of fast-growing fish species and their disease resistance (Lind et al., 2012; Ina-Salwany et al., 2019). Nevertheless, outbreak of many diseases such as Tenacibaculosis (yellow mouth), Streptococcosis and Vibriosis has led to high mortality in fish farms and the industry has suffered huge economic losses (Jantrakajorn et al., 2014; Ina-Salwany et al., 2019; Wynne et al., 2020). The industry has hardly taken steps to selectively breed fishes in order to shape the microbiota as an indicator of health. It has been reported that selective breeding can produce fishes with microbiota that can be manipulated to improve disease resistance (Piazzon et al., 2020).

Currently, there are many genetically improved tilapia and GIFT (Genetically Improved Farmed Tilapia) is the most known breed. Although many studies have employed genetically improved tilapia (Bolivar and Newkirk, 2002; Romana-Eguia et al., 2005; Santos et al., 2013; Mehar et al., 2019), to our knowledge there are only a couple of reports about the microbiome composition in selectively bred fish (Kokou et al., 2018; Brown et al., 2019). In mouse, selective breeding is known to increase the inter-individual gut microbiota similarity (Pang et al., 2012); variation is less in the case of inbred animals compared to their outbred counterparts (Hufeldt et al., 2010). Researchers have also succeeded in producing outbred mice with stable gut microbiota (Hart et al., 2018). Furthermore, the association between the gut microbiome and breeding was studied in mouse models by analysing the effect of the gut microbiome on different breeds (Pang et al., 2012; Kreisinger et al., 2014; Ericsson et al., 2015; Oriá et al., 2018). This link was also explored in plants by examining the impact of the microorganisms on host phenotype (Wagner et al., 2020). Moreover, the microbial taxa that is widespread among the host population is vertically transmitted, and host factors provide them with the optimum ecosystem for colonization (Risely, 2020).

Selective breeding affects host genetic selection, which in turn shapes the gut microbiome (Kokou et al., 2018) that has an important role in, among others, maintaining the host health. The paucity of information regarding the mating strategy-caused changes in fish microbiome that can signal disease propensity led us to examine the differences in the bacteria associated with inbred and outbred Nile tilapia using next-generation sequencing technology.

## Materials and Methods

### Fish Husbandry and Sample Collection

Fertilized eggs (*n*=180) of Nile tilapia, were obtained from wild fish captured from the Nile river, Luxor, Egypt (location GPS: 25°39'56'' N, 32°37'07'' E). These eggs were disinfected with hydrogen peroxide for 10min and placed in egg rockers (Cobalt Aquatics, Rock Hill, South Carolina, United States) installed in a 60L tank with UV treated water, containing 5% NaCl. Around 85% of the eggs were hatched at 28°C within 4days. The hatched larvae were placed in fish transport bags filled with UV treated and 100% oxygen saturated water. These larvae were shipped, within approximately 18h, to the Research Station of Nord University, Bodø, Norway *via* air and their survival rate exceeded 95%. The transported larvae were reared at a maximum density of 27 fish/m^3^ for 5months in a freshwater recirculating system. The rearing conditions were: dissolved oxygen – 100%, water temperature –28°C, photoperiod – LD 13:11. The fish were fed Amber Neptun pellets (0.15–0.8mm, Skretting, Stavanger, Norway) during the rearing period. These fish were designated as the F0 generation and were used for the breeding study.

We randomly chose males and females and produced the inbred and outbred groups. When the fish reached 3,570 degree·days, we anesthetized and PIT-tagged them for tracing the individual families.

Prior to sampling, fish were not fed for 48h. They were sacrificed by immersion in an emulsion containing 12ml of clove oil (Sigma Aldrich, St. Louis, Missouri, United States), 96% ethanol (1:10v/v) and 10L of water (Simões et al., 2011; Konstantinidis et al., 2020). Female fish were used for the study as they are maternal mouthbrooders. Twenty fish each from the inbred and outbred groups were used in this study, and three body sites (mouth, anterior and posterior intestine) of female Nile tilapia were targeted for examining the bacterial communities. Mucus samples from the buccal cavity were taken using swabs (Copan Italia, Brescia, Italy), which were transferred to cryotubes and immediately frozen in liquid nitrogen. Then, the same fish were aseptically dissected to collect the anterior and posterior intestine. The intestine samples were also transferred to cryotubes and snap-frozen in liquid nitrogen. The collected samples were stored at −80°C until further use.

### DNA Extraction for Whole-Genome Sequencing

DNA was extracted from fast muscle using DNeasy Blood and Tissue Kit based on the guidelines provided by the manufacturer (Qiagen, Hilden, Germany). The Invitrogen Qubit 3.0 fluorometer (ThermoFisher Scientific, Waltham, Massachusetts, United States) was used to quantify the concentration of DNA in the samples. Quality (based on 260/280 and 260/230 absorbance ratios) and integrity (based on DIN values) of the extracted DNA samples were checked using Nanodrop 1000 Spectrophotometer (ThermoFisher Scientific) and TapeStation 2200 DNA screen (Agilent Technologies, Santa Clara, California, United States), respectively.

### DNA Extraction for 16S Amplicon Analysis

All the procedures mentioned here were performed under sterile conditions. Before extracting the DNA, intestine samples were transferred to a sterile Petri dish and placed on a cool-pack on dry ice. The intestine was opened and transferred to a 5ml tube containing 1.4mm Zirconium oxide beads (Cayman Chemical, Ann Arbor, Michigan, United States) and 2ml of InhibitEX buffer (Qiagen). Thereafter, DNA was extracted immediately using QIAamp DNA stool Mini Kit (Qiagen) according to the manufacturer’s protocol. The final elution volume was 75μl (ATE buffer). The same extraction method was employed for the mouth samples. The quality and quantity of the extracted DNA were checked with NanoDrop spectrophotometer ND-8000 (ThermoFisher Scientific).

### Libraries Preparation and Sequencing

#### Whole-Genome Sequences

The Nextera DNA Flex library preparation kit with dual indices was used to prepare whole genome libraries based on the manufacturer’s protocol (Illumina, San Diego, California, United States). After tagmentation of the extracted gDNA samples using Bead-linked transposomes at 55°C for 15min, the sheared and tagmented gDNA was washed at 30°C for 15min. Amplification of the tagmented gDNA was performed using a 5-cycle PCR programme wherein the index 1 (i7) and index 2 (i5) adapters were added for sequencing cluster formation. The PCR program was started with an incubation at 68°C for 3min and a subsequent pre-denaturation at 98°C for 3min. In the following step, 5cycles of denaturation at 98°C for 45s, annealing at 62°C for 30s and extension at 68°C for 2min were first performed, followed by a final extension at 68°C for 1min. In the final step of the library preparation, the amplified libraries were purified through a double-sided bead (Bead-linked transposome; Illumina) purification procedure. The quality and normality of the libraries were assessed with the Agilent Tapestation instrument using High Sensitivity D1000 screen tape. After normalization based on the minimum observed molarity, the barcoded samples were pooled before the sequencing run. The 75bp paired-end sequencing was done on a NextSeq 500 sequencer (Illumina) at the sequencing platform of Nord University.

#### Bacterial 16S Sequences

Under sterile conditions, 16S rRNA gene libraries were constructed from DNA extracts using the specific bacterial primers 341F (5'CCTACGGGNGGCWGCAG 3') and 805R (5'GACTACNVGGGTWTCTAATCC 3'; Klindworth et al., 2013) flanked by overhang Illumina adapters targeting the hypervariable V3–V4 region (~460bp). PCR reactions were performed for each sample in 25μl, using Q5® High-Fidelity 2X Master Mix (New England Biolabs, Ipswich, Massachusetts, United States) and 2.5μl of DNA template (5ng/μl). PCR conditions consisted of an initial denaturation step at 95°C for 10min (1cycle), 30cycles at 95°C for 30s, 57°C for 30s, 72°C for 1min, and a final extension step at 72°C for 7min (1cycle).

An agarose gel (1.5%) was employed to check the amplified products. The PCR products were purified using the CleanNGS system (CleanNA, Waddinxveen, Netherlands) following the manufacturer’s instructions. The purified product was subjected to a second PCR (8cycles, 16S Metagenomic Sequencing Library Preparation, Illumina); this step was done to add dual indices and Illumina sequencing adapters Nextera XT Index Primer (Illumina). CleanNGS (CleanNA) was used to purify the obtained amplicon libraries. The quality of the libraries was checked on a Tapestation 2200 platform (Agilent Technologies). Thereafter, the libraries were quantified using the Quant-IT PicoGreen dsDNA assay kit (ThermoFisher Scientific) by the Synergy2 microplate reader (Biotek, Winooski, Vermont, United States). Next, the pooled libraries were quantified using the KAPA Library quantification kit (Roche, Basel, Switzerland). The libraries were checked by realtime qPCR LightCycler 480 (Roche) and then sequenced on an Illumina® MiSeq (PE300) platform (MiSeq Control Software 2.5.0.5 and Real-Time Analysis software 1.18.54.0).

### Sequence Analysis

#### Whole-Genome Sequences

In order to perform demultiplexing and obtain the fastq files, the Illumina Experiment Manager v1.18.1 along with bcl2fastq v2.20.0.422 was used. Thereafter, dual adapter indexes and Ns from the 3' end of the raw reads were trimmed and the quality of the cleaned fastq files was assessed employing Trime_galore v0.4.4 (Babraham Bioinformatics; http://www.bioinformatics.babraham.ac.uk/projects/trim_galore/). The clean reads were then aligned to the reference genome O_niloticus_UMD_NMBU, GCA_001858045.3 (Conte et al., 2017) using Bowtie2 v0.12.8 with the --very-sensitive option (Langmead and Salzberg, 2012). The bcftools pipeline was applied for variant calling (Li, 2011), and the generated SAM files were converted to the binary format and sorted based on coordinates using samtools v1.9. Also, the samtools markedup command was used to mark duplicate reads. Then variants were called using bcftools mpileup command (bcftools 1.9) with the minimum base and mapping quality of 20 (−q 20 −Q 20). Using bcftools filter command accompanied by the options --SnpGap 5 -i ‘MQ>20 and QUAL>20 and DP>100 and DP<450 and TYPE=“snp,” only SNP variants were kept in the Variant Call Format (VCF). The missing genotypes were imputed using imp-states=1,600 option in Beagle v5.0 (Browning and Browning, 2016). Thereafter, using vcftools, the non-biallelic SNP variants were omitted so that the generated VCF file had only the biallelic SNPs (Danecek et al., 2011). This VCF file was read by vcfR package (Knaus and Grünwald, 2017).

#### Bacterial 16S Amplicon Sequences

The generated reads were truncated at 270bp using VSEARCH (Rognes et al., 2016), and then processed using MICCA pipeline (v1.7.2; Albanese et al., 2015). Sequences with a minimum overlap length of 60bp and a maximum mismatch of 20bp were merged. Next, the forward and reverse primers were trimmed off the merged reads and reads which did not contain the primers were discarded. Thereafter, the sequences with an expected error rate (Edgar and Flyvbjerg, 2015) >0.75 were filtered out and shorter than 400bp sequences were discarded. Filtered reads were denoised using the “*de novo* unoise” method implemented in MICCA, which utilise UNOISE3 algorithm (Edgar, 2016). The denoising method generates amplicon sequence variants (ASVs) which is based on correcting sequencing errors and determining true biological sequences at single-nucleotide resolution. The taxonomic assignment of the representative bacterial ASVs was performed using RDP classifier. The sequences were aligned using the NAST (Desantis et al., 2006) multiple sequence aligner, and a phylogenetic tree was prepared using the FastTree software available in the MICCA pipeline.

### Statistical Analysis of Host Genetic Data

To quantify the genetic diversity of the inbred and outbred groups, we first determined the genetic diversity within members of the crossbred groups, and then the between groups genetic diversity. For this, we quantified the level of heterozygosity, using the population package of the Stacks 2.3b. Next, to assess the level of genetic differentiation based on allele frequencies between different groups, the *F_st_* index was calculated using the StAMPP package (Pembleton et al., 2013). In order to quantify the genetic relationship between the inbred and outbred groups, Nei-based genetic distance between individuals was estimated using poppr (Kamvar et al., 2015) and adegenet (Jombart, 2008) packages and visualized using pheatmap package (Kolde and Kolde, 2015). Then the genetic relationship between the crossbred groups was assessed by PCoA (employing the abovementioned Nei-based genetic distance), also using the ape package (Paradis and Schliep, 2019). PERMANOVA (Permutational Multivariate Analysis of Variance) was performed to decipher the significance of genetic differences between the inbred and outbred groups. To further analyze the population structure of the inbred and outbred groups, admixture analysis was performed in adegenet for values of ancestries (K) from 1 to 10 with 10 repeats for each value of K, decided based on Bayesian Information Criteria. Four samples were removed due to the low quality of sequences.

### Statistical Analysis of 16S Amplicon Data

Statistical analysis was conducted using R (version 3.6.3) software. The packages phyloseq (McMurdie and Holmes, 2013) and vegan (Oksanen et al., 2013) were employed to analyse the data. All plots were made using ggplot2 package (Wickham, 2011).

To understand the differences between the proportions of different bacteria in the inbred and outbred groups, we performed chi-square test and the associated *post hoc* analyses. A subset of the most dominant phyla was employed for this analysis. The similarities/differences in α-diversity were checked by Wilcoxon rank-sum test. Bacterial β-diversity was determined using unweighted and weighted UniFrac distances (Lozupone and Knight, 2005). Differences between the bacterial communities of the two groups were visualized by PCoA. After checking the dispersions within the data set of each group, statistically significant differences between the groups were assessed using PERMANOVA (Anderson, 2001; with 9,999 permutations), implemented in adonis function of the vegan R-package (Oksanen et al., 2013). DESeq2 (Love et al., 2014) package was employed to detect the differentially abundant ASVs in the non-rarefied data (McMurdie and Holmes, 2014). The core microbiota was analysed using the packages microbiome and microbiome utilities; at a detection level of 0.2% and prevalence level of 90%. The differences in the core bacterial community in the two crossbred groups were analysed by performing PERMANOVA on weighted and unweighted UniFrac distances. The abundances in the different ASVs which made up the core microbiome were analyzed using Spearman test (Zar, 2014) and correlation plot package (Wei and Simko, 2017).

## Results

### Genetic Background-Associated Changes in the Microbiome of Nile Tilapia

A total of 11,578,530 SNPs were obtained after the initial SNP calling. Bcftools was employed to first calculate genotype likelihoods for each position and then filter out every position with actual sequence variant. Thus, 4,693,720 SNPs were filtered out and finally after biallelic filtration, 6,825,083 SNP variants with an average coverage of 1.74 per sample were used in the final VCF file.

The genetic diversity analysis based on nucleotide sequences revealed that the observed heterozygosity (Ho) values were slightly higher compared to the expected heterozygosity values (He; Table 1).

**Table 1:** Observed (Ho) and expected (He) heterozygosity of the crossbred female Nile tilapia.

	*Ho*	*He*
Outbred-S3	0.171	0.157
Inbred-S1	0.164	0.153
Inbred-C6	0.167	0.155
Outbred-C9	0.166	0.149

The fixation index (*F_st_*) value within groups was 0.04 for both Inbred-S1 vs. Inbred-C6 and Outbred-S3 vs. Outbred-C9 comparisons. On the other hand, the *F_st_* values between crossbred groups were in the range 0.06–0.08 (Table 2).

**Table 2:** Genetic differentiation, based on *F_st_* index, of the crossbred female Nile tilapia.

	Outbred-S3	Inbred-S1	Inbred-C6	Outbred-C9
Outbred-S3	-			
Inbred-S1	0.061	-		
Inbred-C6	0.077	0.041	-	
Outbred-C9	0.044	0.058	0.074	-

**Figure 1:**
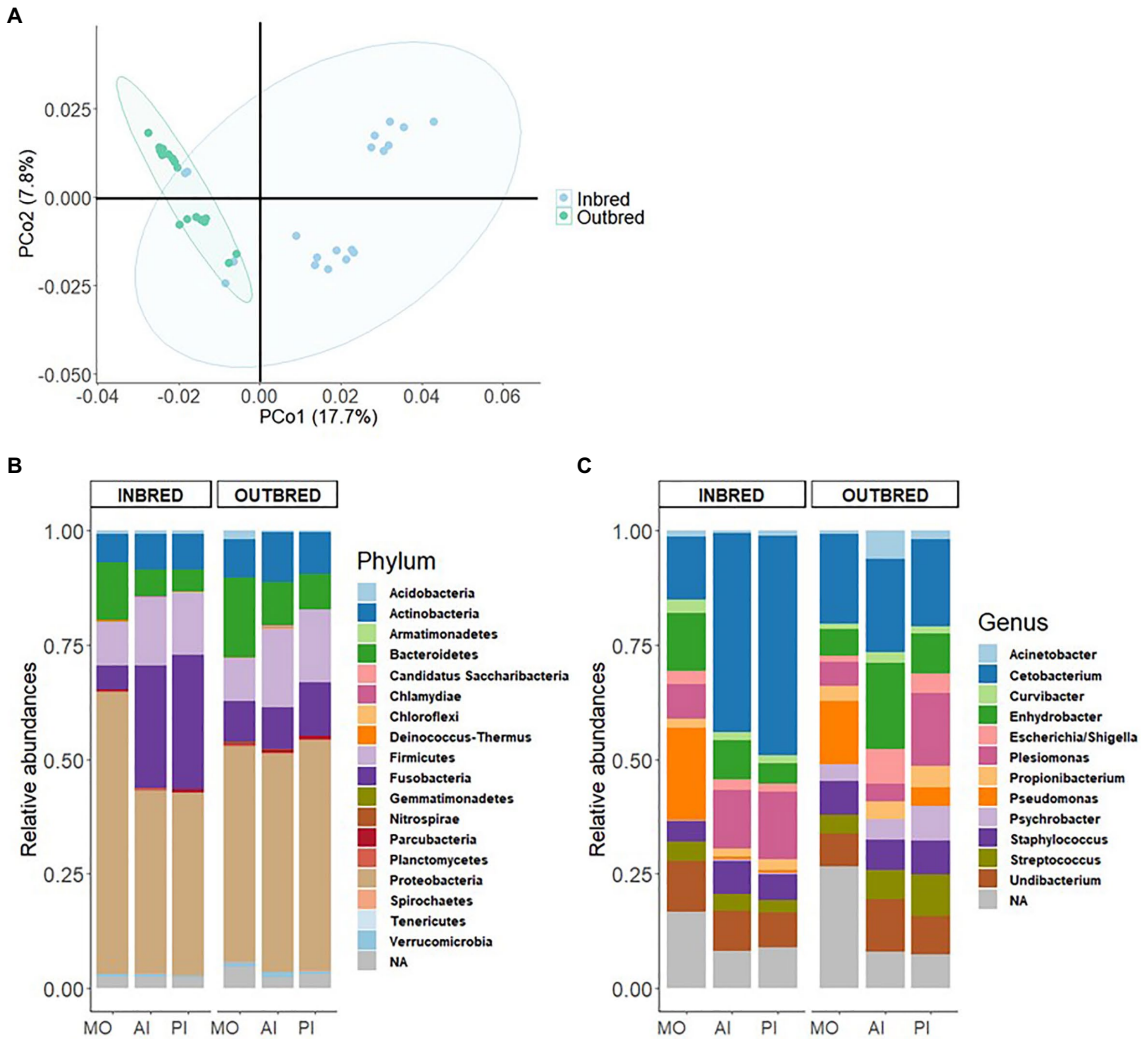
Genetic differentiation and microbiome in the inbred and outbred groups of Nile tilapia. **(A)** Principal coordinates analysis (PCoA) plot based on 6,825,083SNPs of the inbred and outbred groups. The ellipses were generated assuming that the data are from a multivariate normal distribution. **(B)** Phylum-level relative abundance of the microbial composition in the inbred and outbred groups. **(C)** Relative abundance of top 12 genera in the inbred and outbred groups.

The Nei-based genetic distances between the inbred and outbred groups were employed to generate a heatmap to understand their genetic relationships; differences between the groups are seen in Supplementary Figure 1. Principal Coordinates Analysis (PCoA) based on the Nei-based genetic distance indicated that the first two components captured 17.7 and 7.8% of the variation in the data set (Figure 1A). Furthermore, a PERMANOVA test based on the same genetic distance showed that the inbred and outbred groups were significantly different (*p*=0.001). The genetic sub-population clustering based on admixture analysis revealed that K=2 was the optimal number to explain the genetic structure of the inbred and outbred groups (Supplementary Figure 2). The results also indicated that 4 inbred individuals are genetically similar to the outbred population.

To delineate the effect of genetic selection on gut microbiota composition, the inbred and outbred Nile tilapia were reared in a common garden and the environmental and nutritional factors that affect the microbiota were kept constant throughout the experimental period. The amplicon library of 16S rRNA gene, generated 12,034,190 high-quality reads with an average coverage of 54,016 reads per sample. Due to the variation in sample size, the reads were rarefied to 18,000 reads per sample (without replacement). Out of the 120 samples, six libraries with a number of reads below the cut off were discarded. After normalization we obtained 14,228 ASVs, distributed among 30 phyla and 695 genera.

First, we investigated the dominant communities in the two groups. In their order of dominance, the most dominant bacterial phyla in both the inbred and outbred fish groups were *Proteobacteria*, *Fusobacteria*, *Firmicutes*, *Bacteroidetes*, and *Actinobacteria* (Figure 1B). This order of dominance was reflected in the microbial composition at the genus level also. Most of the dominant genera in the two crossbred groups belonged to the phylum *Proteobacteria* (*Acinetobacter*, *Curvibacter*, *Enhydrobacter*, *Escherichia*/*Shigella*, *Plesiomonas*, *Pseudomonas*, *Psychrobacter*, and *Undibacterium*). The most abundant genus was *Cetobacterium* which belongs to the phylum *Fusobacteria* (Figure 1C).

To understand the differences in proportions of the dominant communities in each study group, we performed chi-square test. The analyses revealed that the abundances of the most dominant phyla in both the inbred and outbred groups were significantly different (Supplementary Table 1).

**Figure 2:**
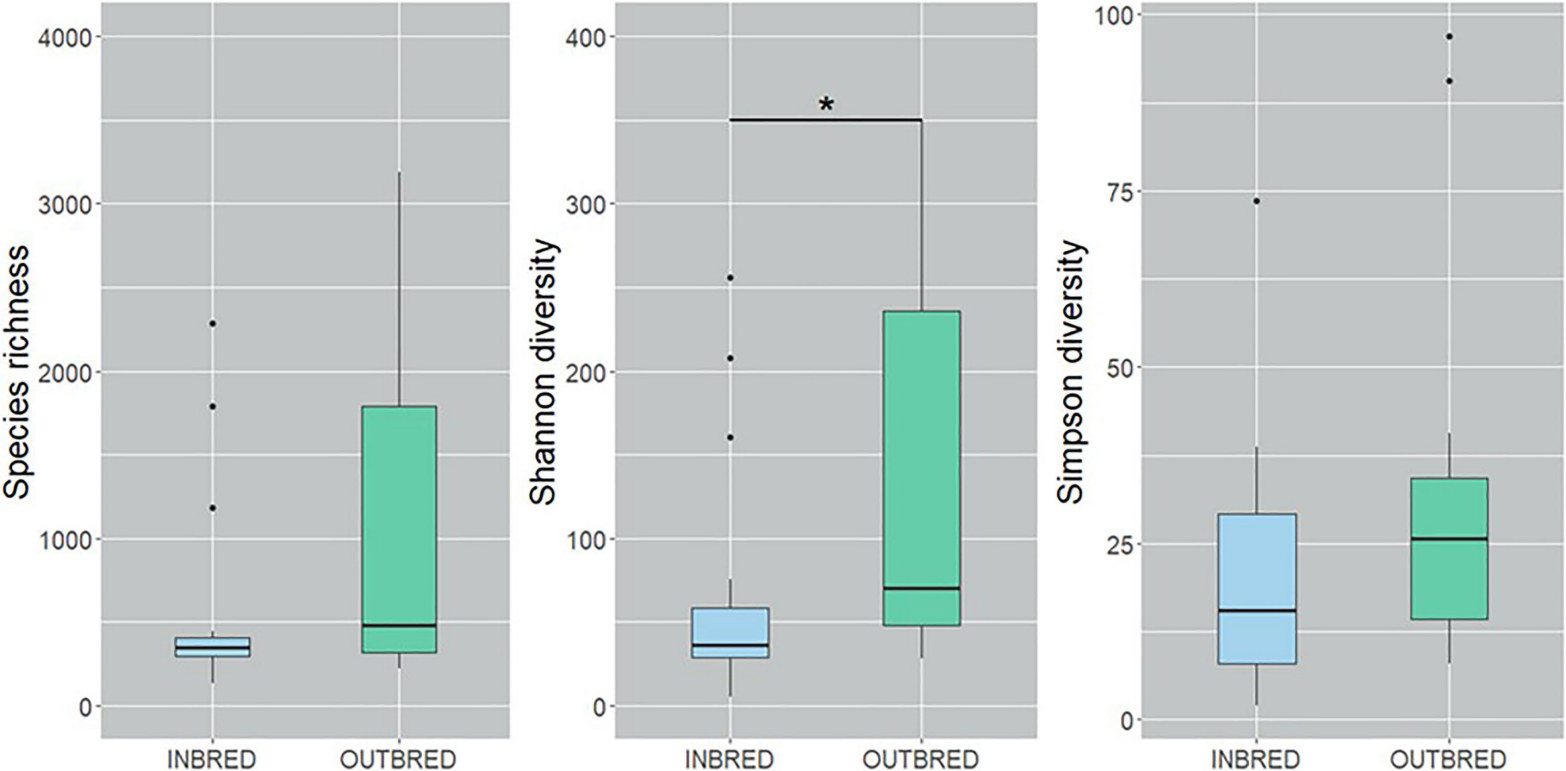
Alpha diversity of the bacteria in the mouth of the inbred and outbred groups of Nile tilapia. Species richness of the groups is not significantly different. Shannon diversity is higher in the outbred group (*p*=0.007, indicated with an asterisk). Simpson diversity indicated an increasing trend in the dominant ASVs of the outbred group (*p*=0.08). The boxplots show minimum, lower quartile, median, upper quartile and maximum values.

**Figure 3:**
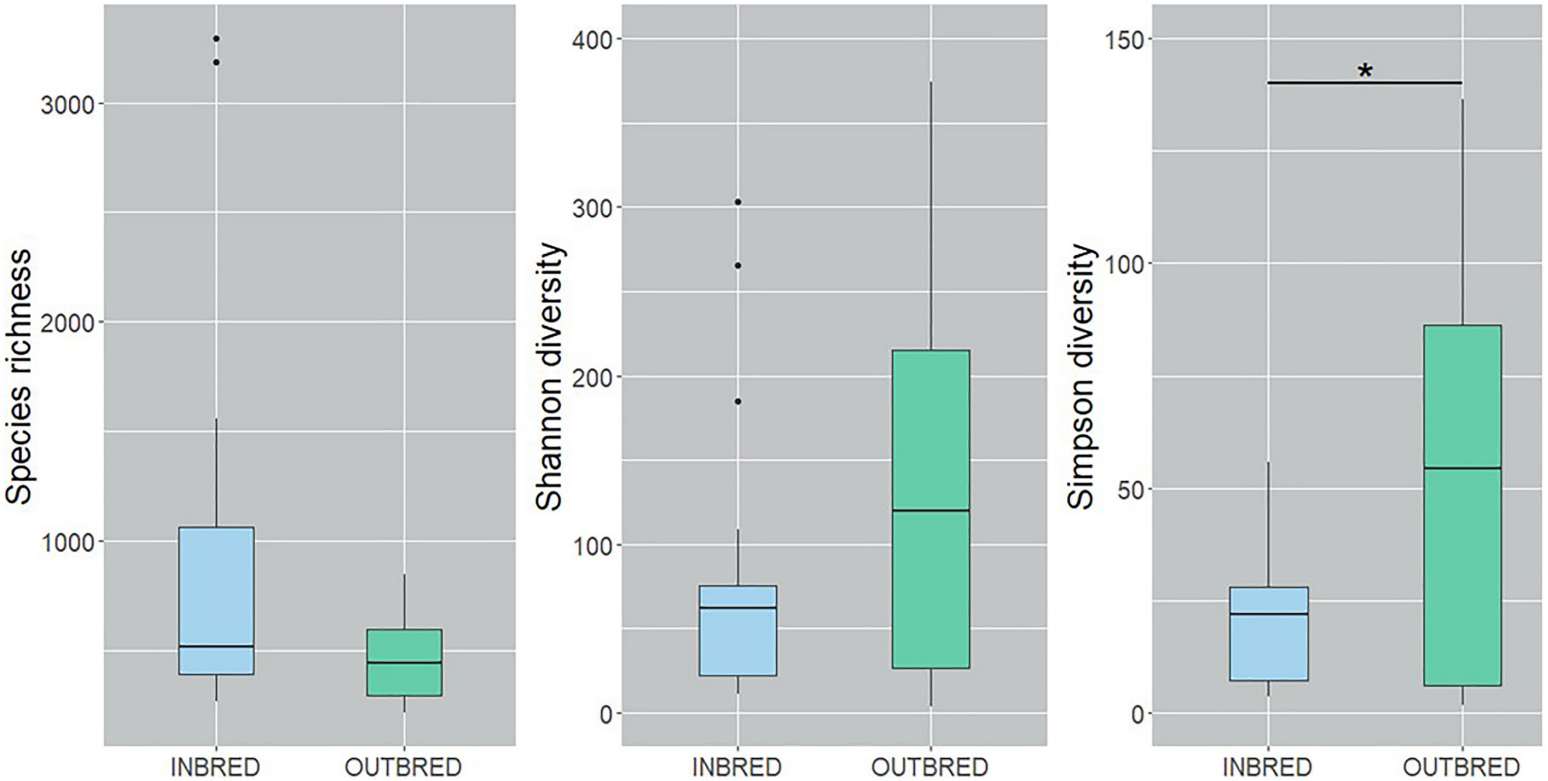
Alpha diversity of the bacteria in the posterior intestine of the inbred and outbred groups of Nile tilapia. Simpson diversity analysis showed that the dominant ASVs are higher in the outbred groups (*p*=0.04, indicated with an asterisk). The boxplots show minimum, lower quartile, median, upper quartile and maximum values.

**Figure 4:**
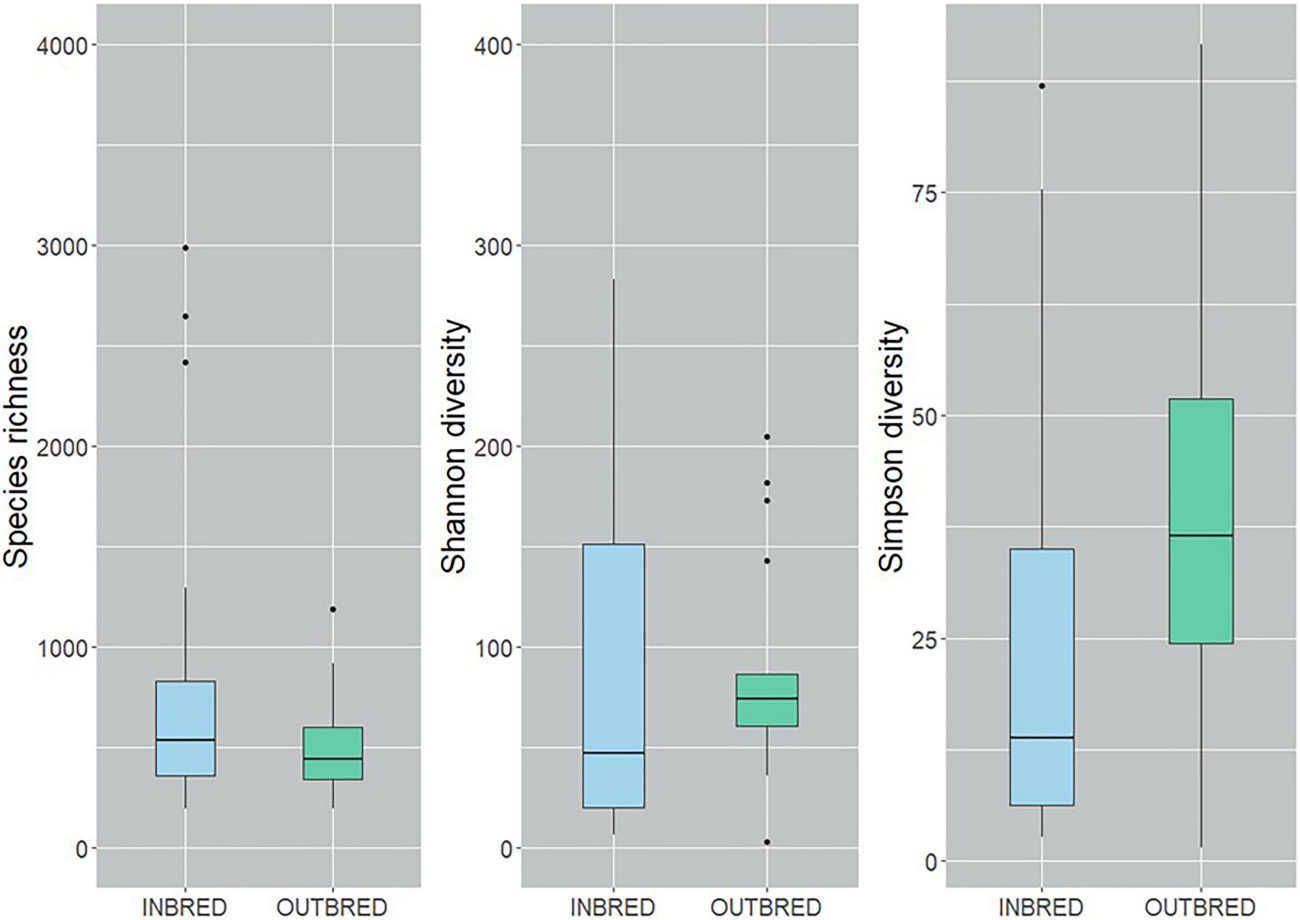
Alpha diversity of the bacteria in the anterior intestine of the inbred and outbred groups of Nile tilapia. There is an increasing trend in the species richness of the inbred group (*p*=0.07). Simpson diversity shows an increasing trend in the dominant ASVs of the outbred group (*p*=0.06). The boxplots show minimum, lower quartile, median, upper quartile and maximum values.

To characterize the microbial diversity within the samples, we calculated three ecological indexes, namely the Chao1 estimator of the number of species, which is a measure of richness, the Shannon diversity which measures the evenness of the microbial populations and the Simpson diversity, which measures the importance of dominant species (Marcon and Hérault, 2015; Hsieh et al., 2016). Shannon diversity analysis showed that the microbial diversity in the mouth of the inbred group was lower compared to the outbred group (Figure 2, *p*=0.01). The Simpson diversity analysis indicated that there were fewer dominant ASVs in the posterior intestine of the inbred group (Figure 3, *p*=0.04). Although there were no significant differences in species richness of the communities associated with the two groups, in each body site (Supplementary Table 2), there was an increasing trend (*p*=0.08; inbred higher richness) in the case of the anterior intestine (Figure 4). Furthermore, the diversity analysis of dominant bacteria (Simpson diversity) in the mouth and anterior intestine revealed a trend in differences (*p*=0.08 and 0.06, respectively; Figures 2, 4).

**Figure 5:**
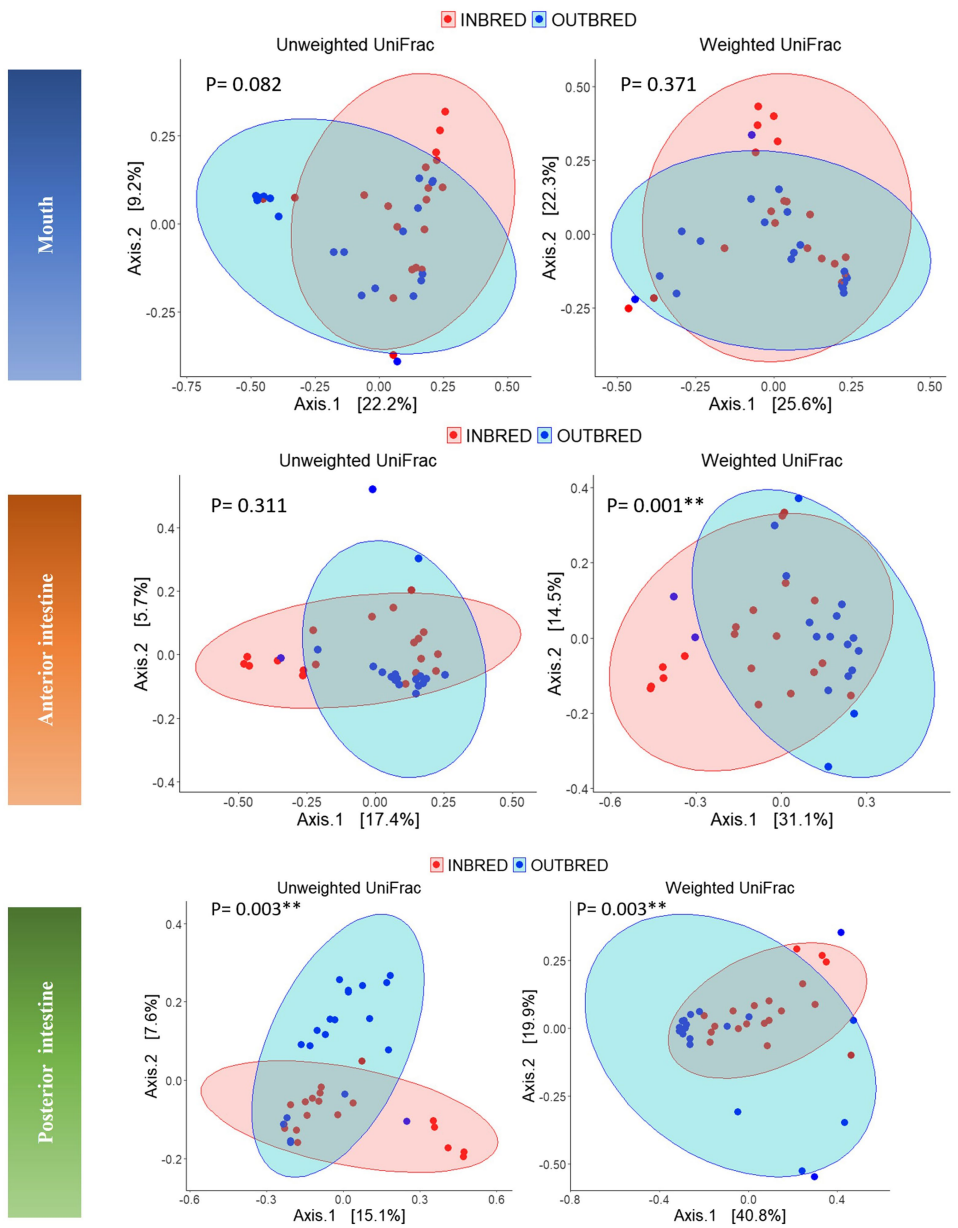
Principal coordinates analyses (PCoA) using unweighted and weighted UniFrac distance matrices of the bacteria in the different body sites of the inbred and outbred groups of Nile tilapia. The ellipses were generated assuming that the data are from a multivariate normal distribution.

Beta diversity analysis was performed to evaluate the overall dissimilarity between the two crossbred groups (Figure 5). The results of PERMANOVA on the unweighted UniFrac distances showed a significant difference between the bacterial composition in the posterior intestine of the inbred and outbred groups (*p*=0.003). There was no significant difference between the communities in the mouth or the anterior intestine of the two groups (*p*=0.082 and 0.311, respectively). In the mouth, there may exist a difference in composition between the two groups, based on the observed trend (Table 3).

**Table 3:** Results of the analysis of homogeneity of group dispersions and PERMANOVA using distance (unweighted and weighted UniFrac) matrices.

		Unweighted UniFrac distance			Weighted UniFrac distance		
Comparison	Variable	*p*-value dispersions	*R* ^2^	*p*-value adonis	*p*-value dispersions	*R* ^2^	*p*-value adonis
Outbred vs. Inbred	Mouth	0.86	0.08	0.08	0.62	0.029	0.37
	Anterior intestine	0.20	0.03	0.31	0.27	0.14	0.001^**^
	Posterior intestine	0.60	0.05	0.003^**^	0.05	0.10	0.003^**^

** Indicates *p* <0.05.

Considering the weighted UniFrac distance, there was a significant difference in the community composition of the anterior intestine (*p*=0.001). In addition, there was a significant difference in the community of posterior intestine (*p*=0.003), but not in the case of mouth (*p*=0.37; Table 3).

### Differential Abundance of ASVs: Outbred Group vs. Inbred Group

The package DESeq2 was used to identify the ASVs with a significantly different abundance in the outbred group compared to the inbred group. In the mouth, the bacteria belonging to *Actinobacteria*, *Armatimonadetes*, *Firmicutes*, and *Proteobacteria* were differentially abundant. There were six genera that belonged to the phylum *Proteobacteria*. Bacteria belonging to two genera (*Psychrobacter* and *Polaromonas*) were 5-fold higher in the outbred group, while those of *Pseudomonas* and *Acinetobacter* were 20-fold higher in the same group. Furthermore, an ASV of the genus *Limnohabitans* was about 9-fold lower and *Comamonas* was 20-fold lower in the outbred group. *Lachnospiracea_incertae_sedis* were about 5-fold higher in the outbred group, whereas the genus *Bacillus* was 20-fold lower. These two genera belong to the phylum *Firmicutes*. Also, *Armatimonadetes_gp5* was 20-fold lower in the outbred group (Supplementary Figure 3). In the anterior intestine, the majority of the ASVs that were differentially abundant in the outbred group had fold changes between −5 and−15 (Supplementary Figures 4, 5) compared to the inbred group. However, the fold changes of the differentially abundant ASVs of *Bacteroidetes*, *Fusobacteria*, and *Proteobacteria* in the anterior intestine were between −5 and−10 (Supplementary Figure 4 shows selected differentially abundant ASVs; Supplementary Figure 5 shows all the differentially abundant ASVs). Similarly, in the posterior intestine, out of 31 ASVs that were differentially abundant, 30 ASVs had fold changes between −5 and−28 in the outbred group, while only one ASV that belongs to *Acinetobacter* was 20-fold higher in the outbred group (Supplementary Figure 6). Moreover, ASVs of *Pediococcus* and *Bifidobacterium* which belong to *Firmicutes* and *Actinobacteria*, respectively, were lower (log fold change; −5 and−8, respectively) in the posterior intestine of the outbred group (Supplementary Figure 6).

### Core Microbiome and Variability in Taxa

**Figure 6:**
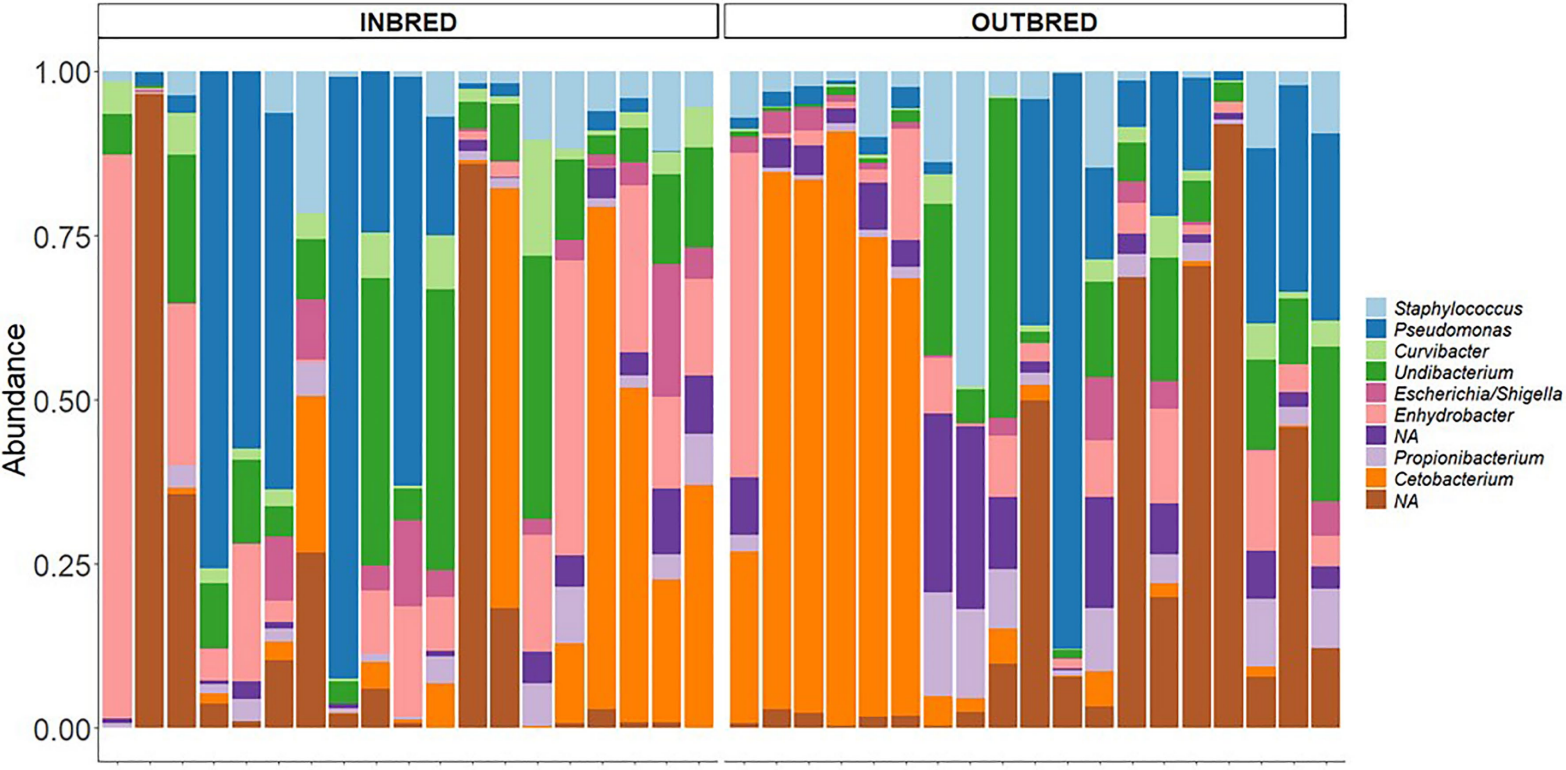
Core microbiota in the mouth of the inbred and outbred groups of Nile tilapia. NAs: Not classified at the genus level, but at the order level, they are classified as *Actinomycetales*, *Sphingobacteriales*, in both groups.

**Figure 7:**
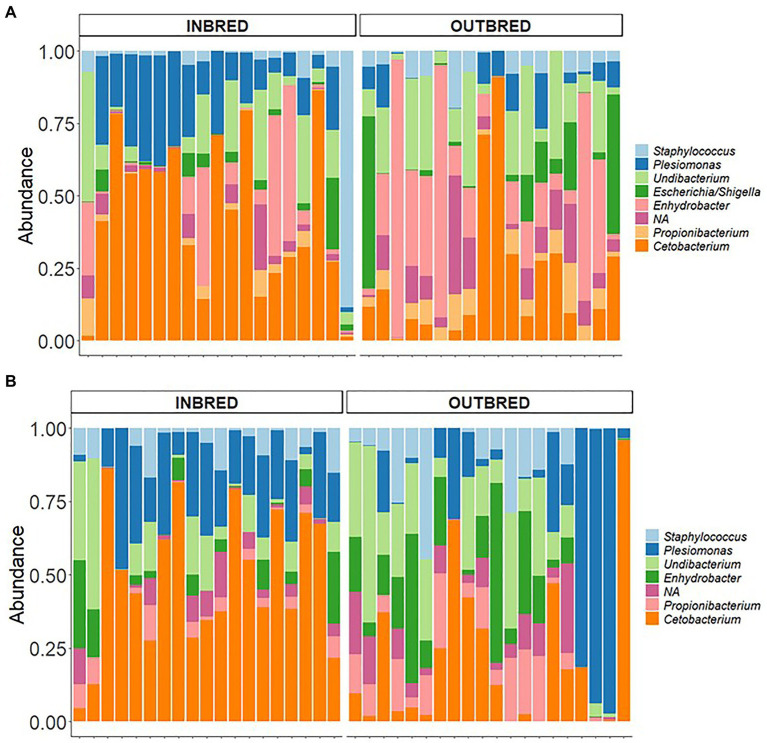
Core microbiota in the anterior and posterior intestine of the inbred and outbred groups of Nile tilapia. **(A)** anterior intestine and **(B)** posterior intestine. NA: at the order level is classified as *Actinomycetales*.

In the mouth, 9 ASVs of the core microbiota belonged to the genera *Staphylococcus*, *Curvibacter*, *Undibacterium*, *Escherichia*/*Shigella*, *Enhydrobacter*, *Propionibacterium*, and *Cetobacterium*. However, two bacteria were classified only up to the order level – *Actinomycetales, Sphingobacteriales* (Figure 6). Taking all the 9 ASVs together, we observed a significant difference in the core microbiome in the inbred and outbred groups; only for unweighted UniFrac distance (*R*^2^=0.073, *p*=0.043; weighted UniFrac distance showed no significant difference; *R*^2^=0.024, *p*=0.445; Table 4). In the anterior and posterior intestine, the core ASVs were *Staphylococcus*, *Plesiomonas*, *Undibacterium*, *Enhydrobacter*, *Propionibacterium*, and *Cetobacterium* (Figures 7A,B). One extra genus was a member of the core microbiota in the anterior intestine (*Escherichia*/*Shigella*). One ASV in the anterior and posterior intestine was not classified up to the genus level, but was annotated as *Actinomycetales* (Figures 7A,B). The core microbiota in the anterior intestine of the inbred group was different from that of the outbred group; the weighted UniFrac distances-based assessment indicated the significant difference (PERMANOVA test; *R*^2^=0.155, *p*=0.001) between the two crossbred groups. As for the posterior intestine, we cannot specify that there is a significant difference between the crossbred groups (Table 4). The inter-individual variation in the abundance of the core microbiota in the intestine samples of the inbred group was less pronounced compared to the outbred groups (Supplementary Figure 7). On the other hand, the inter-individual variation in the abundances was more pronounced in the mouth of the inbred compared to the outbred group (Supplementary Figure 8).

**Table 4:** Results of the analysis of homogeneity of group dispersions and PERMANOVA using distance (unweighted and weighted UniFrac) matrices of the core microbiota.

		Unweighted UniFrac distance			Weighted UniFrac distance		
Comparision	Variable	*p*-value dispersions	*R* ^2^	*p*-value adonis	*p*-value dispersions	*R* ^2^	*p*-value adonis
Outbred vs. Inbred	Mouth	0.834	0.0734	0.043^*^	0.742	0.0241	0.445
	Anterior intestine	0.08	0.0355	0.352	0.323	0.1553	0.0011^**^
	Posterior intestine	0.208	0.0181	0.541	0.003^**^	0.1238	0.0025^**^

* Indicates *p* <0.05 and

** indicates *p* < 0.01.

## Discussion

The genetic structure of wild/domestic/experimental animals can be altered through breeding to retain desired phenotypic and genotypic traits across generations. It is known that selective breeding can preserve desired traits, which can affect the bacterial profile that is highly correlated to host health.

Gut microbiota in fish has been studied extensively in recent years considering mainly its importance in host health. In the present study, we used genetically distinct (based on SNP analysis) inbred and outbred Nile tilapia to investigate the impact of crossbreeding on the composition of the mouth and intestine bacteria.

### Mouth and Intestine Bacterial Community Composition and Diversity in the Inbred and Outbred Groups

Although male Nile tilapia are widely farmed because of their higher growth rate, in the present study, we analyzed the microbial community in females, which are mouthbrooders. Hence, we believe that studying the microbial communities in its mouth will yield interesting results. In humans, microbiota is transferred from different body sites of mothers to infants (Ferretti et al., 2018). Moreover, microbial symbionts from discus (*Symphysodon aequifasciata*, another fish of the family Cichlidae) parents are vertically transferred to fry through feeding of a cutaneous mucus secretion (Sylvain and Derome, 2017). The most dominant phyla found in our samples were *Proteobacteria*, *Fusobacteria*, *Firmicutes*, *Bacteroidetes*, and *Actinobacteria* (Supplementary Figure 9). These are known to be the most represented phyla in model fishes such as zebrafish and threespine stickleback (Legrand et al., 2020). They are also dominant in farmed fishes like Nile tilapia even though many factors including diet (Ray et al., 2017; Souza et al., 2020), rearing systems (Giatsis et al., 2015; Yukgehnaish et al., 2020), and salinity (Zhang et al., 2016; Yukgehnaish et al., 2020) affect the abundance of these phyla in the gut. However, the role of crossbreeding in shaping microbial communities has not yet been reported in fish although it is studied in mice (Pang et al., 2012; Kreisinger et al., 2014), mammals (Alessandri et al., 2019), and plants (Wagner et al., 2020).

The dominant phyla were the same in both the inbred and outbred groups of Nile tilapia. *Proteobacteria* are facultative anaerobes, and they are the most abundant bacterial phylum in fish gut (Egerton et al., 2018). Furthermore, bacteria such as *Escherichia* and *Enhydrobacter* belonging to this phylum have the ability to make the gut environment conducive to strict anaerobes which colonize healthy gut (Shin et al., 2015). Although the aforementioned genera were present in the mouth and intestine of both the outbred and inbred fish, their abundances in the two groups were different. In addition, the genus *Curvibacter* which was present in both groups is known to have a critical role in colonization in freshwater invertebrates (Wein et al., 2018).

Alpha diversity analysis revealed that our crossbreeding strategy increased the microbial evenness in the mouth of the outbred group, in which we observed apparently higher species richness. The increasing trend in the dominant bacteria in the mouth and the anterior intestine of the outbred group along with the significant increase in the posterior intestine suggests that the dominant bacteria in the outbred groups are more diverse compared to the inbred group. On the other hand, the increasing trend in the species richness in the anterior intestine of the inbred group suggests that the bacterial community is more diverse in this intestinal segment of the inbred group compared to the outbred group. The abovementioned findings are similar to the results of the PCoA analysis that used UniFrac distances. Microbial diversity is believed to have a positive correlation with host health (Deng et al., 2019). However, Reese and Dunn (2018) have stated that “understanding diversity in host-associated microbial communities will not be as simple as ‘more diversity is better’.” Hence, it is not ideal to correlate host health with the diversity in the outbred group. Studies in Nile tilapia have not reported a significant difference in the diversity of gut microbiota as a pathogenic effect (Suphoronski et al., 2019; Silva et al., 2020). On the other hand, while diet was shown to increase the species richness of bacteria in the gut, another environmental factor, salinity, was found to decrease the richness of bacteria in Nile tilapia (Zhang et al., 2016). The implication of the increasing trend in diversity in the anterior intestine of the inbred group should be clarified by conducting studies on the bacteria in this segment and their effect on nutritional physiology (Hallali et al., 2018). Thus, in addition to the aforementioned factors, we suggest that crossbreeding is a determinant of both the mouth and intestine bacterial diversity in female Nile tilapia.

### Significant Differences Between the ASV Abundance of the Inbred and Outbred Groups

Fish gut harbors complex and diverse microbial communities, and the site is a reservoir of many opportunistic pathogens belonging to the genera *Acinetobacter*, *Aeromonas*, *Psychrobacter*, *Flavobacterium*, *Pseudomonas*, and *Pleisomonas*. Many commensal bacteria including *Cetobacterium*, *Methylobacterium*, *Sphingomonas*, and *Propionibacterium* (Suphoronski et al., 2019; Legrand et al., 2020; Silva et al., 2020) that colonise the fish gut are essential for the production of vitamin B12 and antimicrobial metabolites (Suphoronski et al., 2019; Legrand et al., 2020), protection against pathogens such as *Flavobacterium* (Boutin et al., 2014), and improving host health (Boutin et al., 2013). The differential ASV analysis revealed that the abundances of some of these opportunistic pathogens (*Psychrobacter*, *Pseudomonas*, and *Acinetobacter*) were more than 5-fold in the mouth of the outbred group compared to the inbred group. In the anterior and posterior intestine of the outbred group, although the opportunistic pathogens belonging to the genera *Acinetobacter*, *Aeromonas*, *Pleisomonas*, *Psychrobacter*, *Pseudomonas*, and *Flavobacterium* were differentially abundant, their fold changes were less than 5-fold. The bacterial community in the mouth is extensively exposed to the external environment, and we found that the opportunistic pathogens in the mouth are more abundant in the outbred group. On the other hand, the abundance of potential pathogens was lower in the intestine of the inbred group. *Pseudomonas* sp. are opportunistic pathogens and they cause high mortality in farmed fishes (Oh et al., 2019). Moreover, bacteria belonging to *Flavobacterium* were reported to cause acute bacteremia primarily in small fishes or more chronic disease in larger fishes (Semple et al., 2020). Although the outbred fish had a more diverse microbiome, they appear to harbor potential opportunistic bacteria also.

Interestingly, the abundance of potential beneficial bacteria (*Cetobacterium*, *Methylobacterium*, *Sphingomonas*, and *Propionibacterium*; Boutin et al., 2013, 2014; Suphoronski et al., 2019; Legrand et al., 2020) was higher in the inbred group. Many studies report that commensal microbiota in the gut plays an important role in regulating the growth of other microbes by competing for space and nutrition. The mouth of the inbred fish had higher abundance of *Aeromonas* sp. which was found to compete for nutrients and play a negative role during infection (Wiles et al., 2016; Legrand et al., 2020). On the other hand, the bacteria that had higher abundance in the posterior intestine of the inbred tilapia, namely *Enhydrobacter* sp., is a commensal microbe in rainbow trout (*Oncorhynchus mykiss*), which is known to produce entericidin, and this antitoxin peptide inhibits the growth of certain pathogens such as those belonging to *Flavobacterium* (Legrand et al., 2020). Furthermore, *Pediococcus* and *Bifidobacterium* which were found to be more abundant in the anterior and posterior intestine of the inbred groups compared to the outbred group are known to outcompete some invasive pathogens, associated with tilapia intestinal mucosa (Ferguson et al., 2010; Standen et al., 2013) and promote fish growth (Ayyat et al., 2014). Thus, the inbred group had a higher abundance of potential beneficial commensal bacteria.

### Changes in Core Microbiome

The transient allochthonous microbiome of fish is associated with digesta and is usually expelled after some period as they are predominantly influenced by diet. On the other hand, the resident microbes that belong to the autochthonous microbiome colonise the mucus surface in the gut and make up the core microbiome (Egerton et al., 2018). These microbial communities, which are known to be vertically transmitted (Risely, 2020), associate with the host’s cells (Egerton et al., 2018; Legrand et al., 2020). In the present study, the core microbiome in each body site was determined based on the ASVs present in all samples in each group. However, the inter-individual variation in abundance that we observed is similar to the learning from studies on zebrafish (Burns et al., 2016) and mice (Pang et al., 2012). In mice, inbreeding was found to reduce the inter-individual variation (Pang et al., 2012). The inter-individual variation in the core microbiome in the intestine of the inbred group is much lesser compared to the outbred group. In contrast, such similarity was not observed in the mouth of the inbred fish; this was attributed to the effect of external environment in other studies (Lokesh and Kiron, 2016; Krotman et al., 2020). However, in the present study, environmental factors were kept constant throughout the study period. In humans, the initial oral colonizers from the vagina and mother’s milk and mouth can be perturbed by environmental factors (Kilian, 2018).

The most dominant bacterial phylum in the two study groups was *Proteobacteria*. Nevertheless, *Cetobacterium* (phylum *Fusobacteria*) was found to be dominant in the anterior and posterior intestine of the inbred group, while its proportion was reduced in the outbred group. Previous studies conducted on Nile tilapia showed that the composition of *Cetobacterium* spp., the most prevalent genera in tilapia gut, was not affected by diets (Ray et al., 2017) or presence of pathogens (Suphoronski et al., 2019; Silva et al., 2020). Other reports that studied the influence of factors including rearing environment (Giatsis et al., 2015), and salinity (Zhang et al., 2016) on the gut microbial composition substantiates our finding that *Cetobacterium* is a core member of the bacterial community. Based on the present study, it appears that the crossbreeding strategy does not impact the presence of this core member in the mouth and intestine of Nile tilapia.

Some of the commonly reported bacteria in the intestine of Nile tilapia (*Staphylococcus*, *Cetobacterium*, *Plesiomonas*, *Enhydrobacter*, *Undibacterium*, and *Propionibacterium*) were present in both groups. However, some core microbiome members such as *Pseudomonas* and *Curvibacter* were present only in the mouth of both groups. A study employing turbot (*Scophthalmus maximus*) showed that a similar microbiome community was present in the intestine of different breeds fed with different diets and reared in different water environments. In addition, it was reported that core microbiome could colonize fish gut for a long term and it could have a vital physiological significance to the host (Zhang et al., 2020). This suggests that fishes preserve their core microbiome community despite differences in environmental factors.

### Host Genetics and Intestine Microbiome

Growing evidence shows that host genetics plays a key role in shaping the gut microbiome of mammals (Hufeldt et al., 2010; Miller et al., 2018; Alessandri et al., 2019), but not to the same degree as that of environmental factors (Davenport, 2016). While there are many reports on diet-based microbiota differences in fish, evidences of fish genetics-associated microbiota are sparse (Li et al., 2014; Kokou et al., 2018).

Our genetic diversity analysis indicated a small but significant difference between the inbred and outbred fish. Unexpectedly, the observed heterozygosity was slightly higher than the expected heterozygosity, probably arising from the low genetic diversity values in both the inbred and outbred groups. The Ho, He, and *F_st_* results that we obtained are likely due to small number of founders with a similar genetic background since the F0 generation of the fish were caught from the same area. The F0 itself may have lost considerable genetic diversity, as noted for birds; a small number of founders in a population increased the probability of inbreeding and associated gene diversity loss (Jamieson, 2011).

Wild Nile tilapia populations in West Africa are reported to have low diversity, especially, the species within a particular region; for example in Gambia River and the far western region of the Niger River (Lind et al., 2019). Nile tilapia is seen as a range-limited species in these areas, and founder effect was reported to be the reason for their genetic diversity reduction (Lind et al., 2019). In addition, *F_st_* results also indicated the low genetic differentiation within the inbred groups as well as the outbred groups.

Anthropogenic needs not only alter species behavior, feeding habits, rearing environment, and traits within the host genotype but also reshape the gut microbiota of domesticated/captivated animals (Li et al., 2014; Alessandri et al., 2019). A study on blue tilapia, which was selectively bred to retain a host genotype, has reported that gut microbiome was linked to host genotype as well as specific bacteria such as *Cetobacterium somerae* (Kokou et al., 2018). This bacterium is a cobalamin producer (Tsuchiya et al., 2008; Degnan et al., 2014) and fishes with high abundance of *C. somerae* do not require dietary vitamin B12 (Sugita et al., 1991; Tsuchiya et al., 2008).

In order to analyse the genetic effect (by controlling the mating strategy) on the mouth and gut microbiota, the fish were kept in the same environmental conditions and fed the same diet, since both these factors are determinants of host microbial communities. Thus, crossing strategy influenced the microbial alpha diversity and composition in Nile tilapia. A similar effect on the midgut microbiota composition was observed in selectively bred trout (Brown et al., 2019). In addition, a study conducted on mice suggested that the alpha diversity of the gastrointestinal tract microbiota is slightly decreased in the inbred individuals (Kreisinger et al., 2014). Thus, the differences in the diversities of the microbial communities of the two groups could be attributed to crossbreeding strategy.

The differences in abundance of the microbial composition of the core microbiome in the individual samples from the mouth of the inbred fish were more pronounced compared to the outbred groups. In the mouth, influence of an external environmental factor (water) appears to surpass that of the host genetics. On the other hand, there was more similarity in the abundance of the bacterial communities in the individual intestine samples of the inbred group compared to the outbred group of Nile tilapia. Host genetics is known to have a long-lasting effect on the gut microbial communities and this is due to maternal transfer during early development (Kreisinger et al., 2014). A core microbiota is heritable in several species (Hauffe and Barelli, 2019), including cichlids (Baldo et al., 2017). The similarities in the abundances of the taxa in the inbred group of Nile tilapia, which is also a cichlid fish, suggest that the microbial composition in the gut is more established without being affected by the external environment. A study conducted in mice showed that the inter-individual variation in the gut microbiome of the inbred group is lower compared to the outbred animals (Hufeldt et al., 2010). Furthermore, in humans the similarity of the gut microbiome is higher among closer relatives in families (Zoetendal et al., 2001). Therefore, this finding suggests that the genetic factor is more prominent in the intestine of the inbred groups and the effect is likely the inheritance of the microbial profile to the offspring of the fish, especially the core microbiome.

We report for the first time the effect of inbreeding and outbreeding on the mouth and intestine microbiome in Nile tilapia. The genetic relationship and structure analysis indicated the genetic differentiation between the inbred and outbred groups. Differential ASV analysis revealed the abundance of the potential opportunistic pathogens such as *Flavobacterium* in the outbred group and beneficial bacteria like *Bifidobacterium* and *Pediococcus* in the inbred group. We also found that *Cetobacterium* is the core member in both groups, but its abundance was higher in the intestine of the inbred group. The inbred fish which has less inter-individual microbiome variability, could be a better choice for controlled studies that examine the maternal transfer of intestine microbiome to offspring. We highlight that crossbreeding can influence Nile tilapia bacterial communities.

## Data Availability Statement

The data used in this study is available at European Nucleotide Archive (ENA) with accession no PRJEB40093. Whole-genome data of the host are available at Sequence Read Archive (SRA) with accession no PRJNA719847.

## Ethics Statement

The animal study was reviewed and approved by Norwegian Animal Research Authority, FOTS ID 1042.

## Author Contributions

VK, JF, and YA designed the study. YA carried out the sampling and lab work, analysed the data, and wrote the manuscript. CD also analysed the data. SL helped in sequencing and data generation. DA was involved in initial data analyses. OJ and AN prepared the whole genome shotgun sequencing libraries. OJ performed the SNPs analysis of the host. YA, JF, CD, and VK interpreted the data. VK, JF, and CD reviewed and edited the manuscript. All authors contributed to the article and approved the submitted version.

## Funding

This study was funded by the European Research Council (ERC) under the European Union’s Horizon 2020 Research and Innovation Programme (grant agreement no. 683210), the Research Council of Norway under the Toppforsk Programme (grant agreement no. 250548/F20), and the Faculty of Biosciences and Aquaculture (Nord University, Norway).

## Conflict of Interest

The authors declare that the research was conducted in the absence of any commercial or financial relationships that could be construed as a potential conflict of interest.

## Publisher’s Note

All claims expressed in this article are solely those of the authors and do not necessarily represent those of their affiliated organizations, or those of the publisher, the editors and the reviewers. Any product that may be evaluated in this article, or claim that may be made by its manufacturer, is not guaranteed or endorsed by the publisher.
