# Supplementary material for: Breeding Strategy Shapes the Composition of Bacterial Communities in Female Nile Tilapia Reared in a Recirculating Aquaculture System
Source: Front Microbiol. 2021 Sep 10;12:709611. doi: 10.3389/fmicb.2021.709611 (PMC8461179; doi:10.3389/fmicb.2021.709611)
Supplement: Supplementary file 1 [file Data_Sheet_1.docx]

**Breeding strategy shapes the composition of bacterial communities in female Nile tilapia reared in a recirculating aquaculture system**

Yousri Abdelhafiz^1^, Jorge M. O. Fernandes^1^, Simone Larger^2^, Davide Albanese^2^, Claudio Donati^2^, Omid Jafari^1,3^, Artem V Nedoluzhko^1^, Viswanath Kiron^1^*


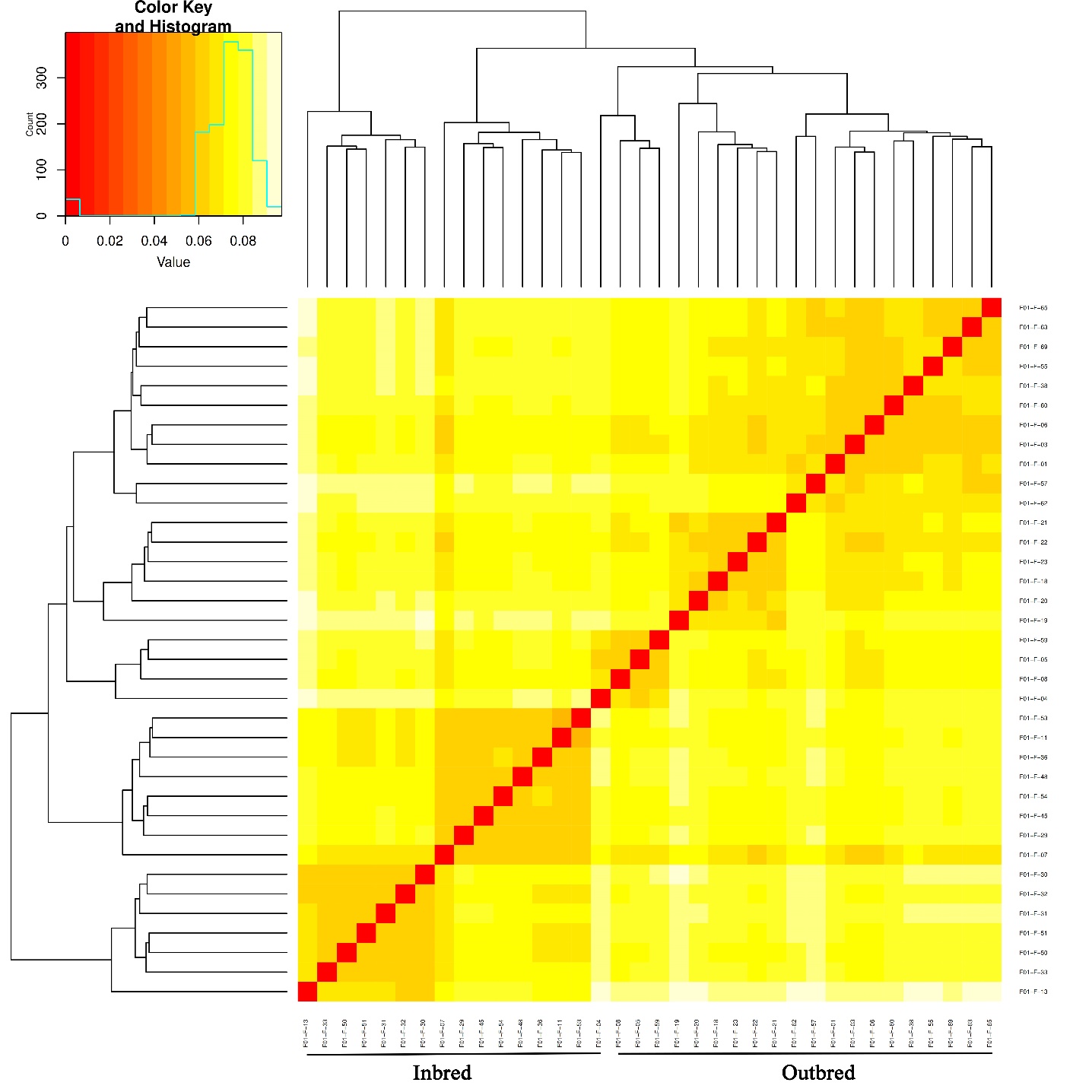


**Supplementary Figure 1.** Unbiased Nei genetic distance clustering based on SNPs from crossbred Nile tilapia groups.


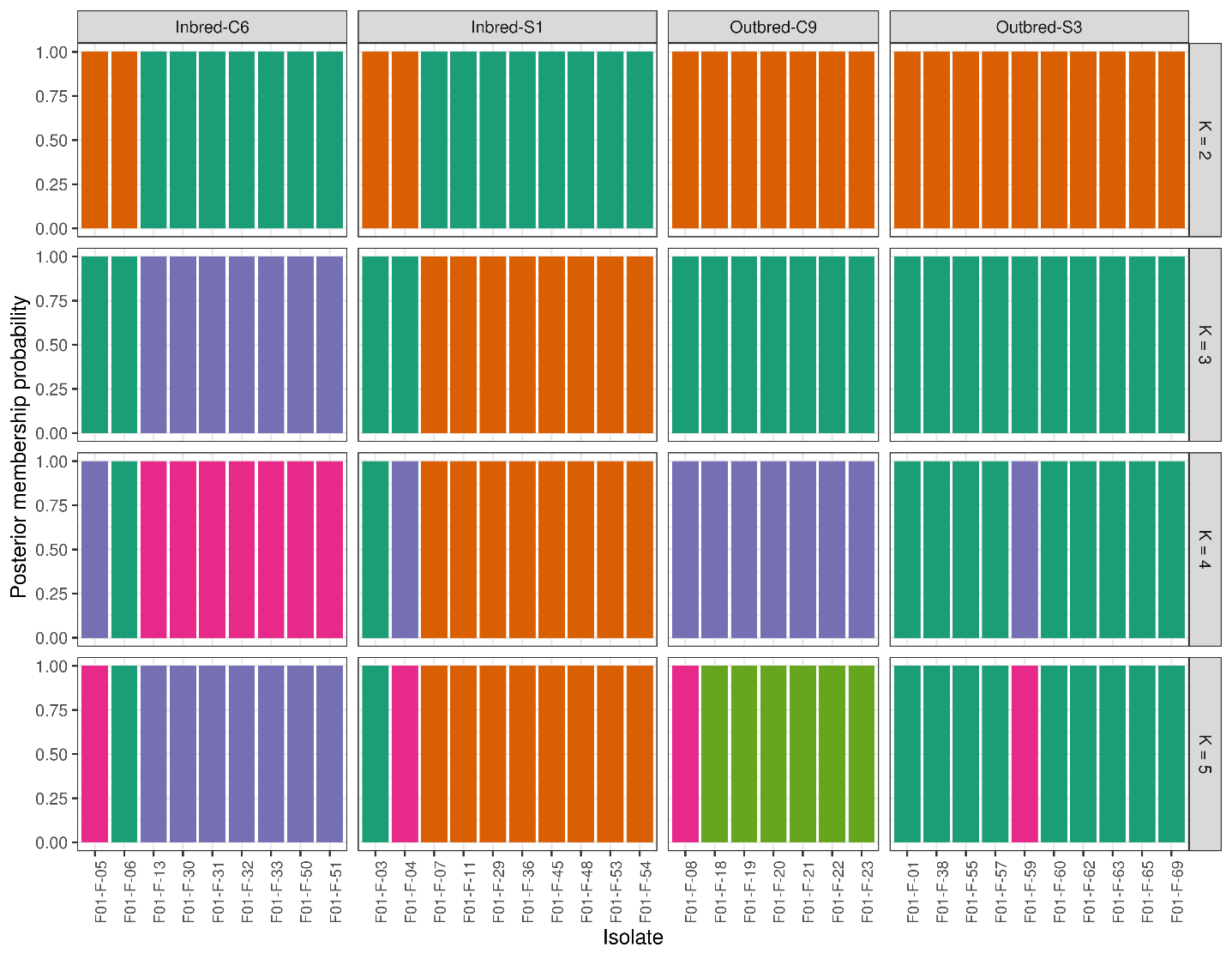


**Supplementary Figure 2.** Genetic structure of the female Nile tilapia individuals, revealed through admixture analysis. Here we explain only the graphs with K=2, which is the optimum at which the inbred and outbred groups are differentiated. Dark orange colour is the outbred group and seagreen is the inbred group. Four individuals from the inbred group are genetically similar to the outbred population.

**
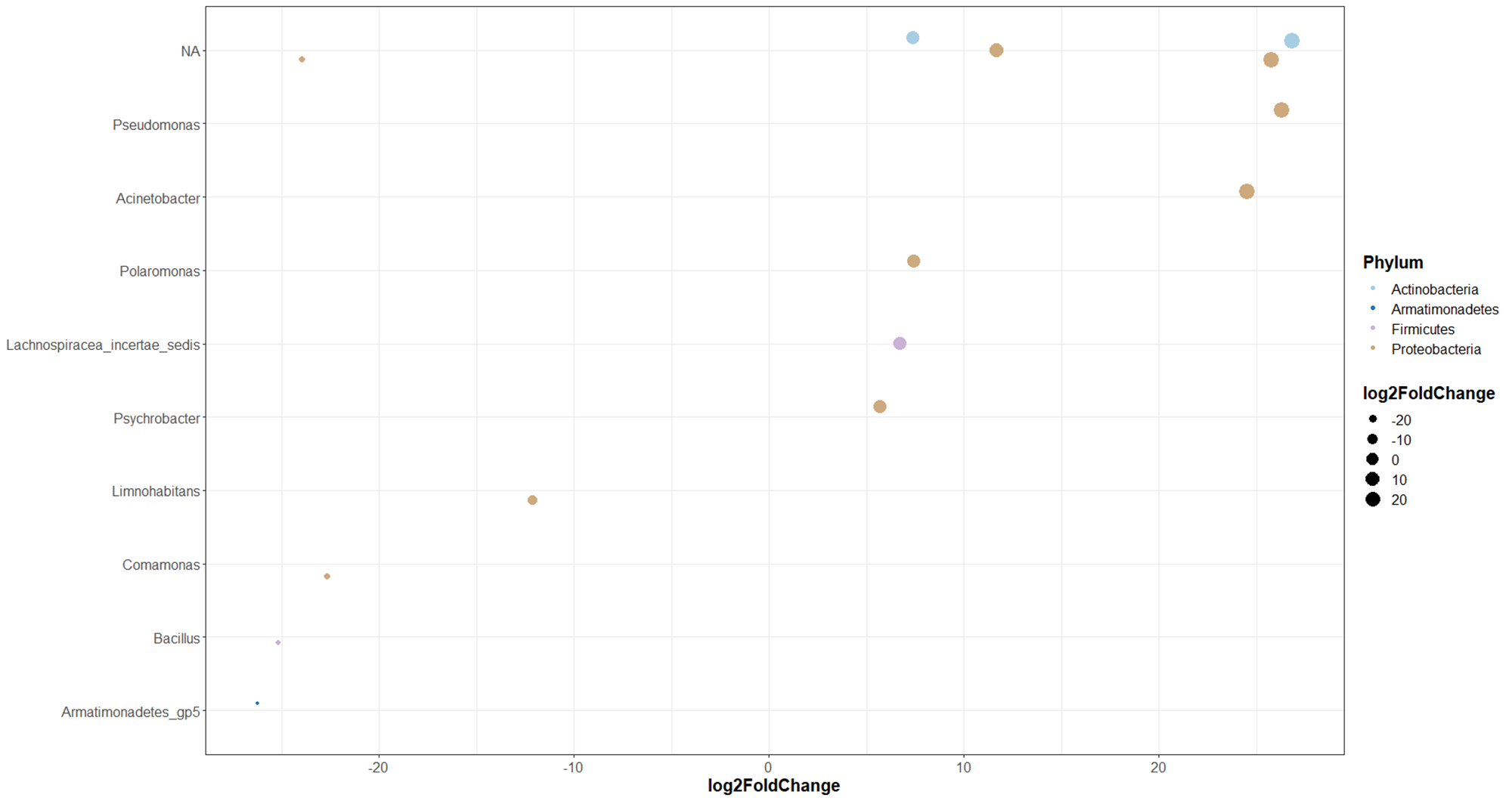
**

**Supplementary Figure 3.** The significantly abundant ASVs in the mouth of the outbred group compared to the inbred group of tilapia. Size of the dots increases with the fold change.

**
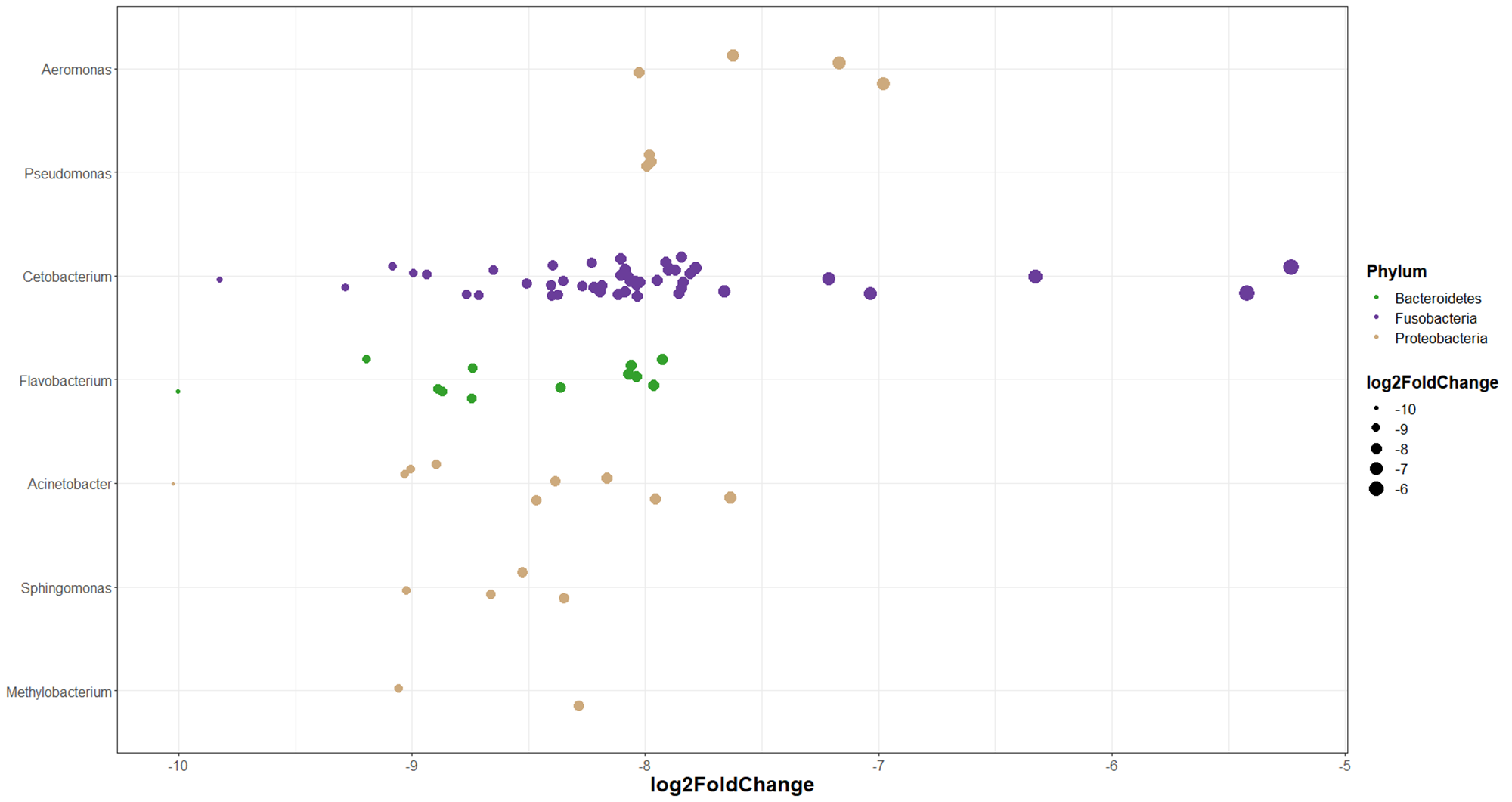
**

**Supplementary Figure 4.** The significantly (selected) abundant ASVs in the anterior intestine of the outbred group compared to the inbred group of tilapia. Size of the dots increases with the fold change.


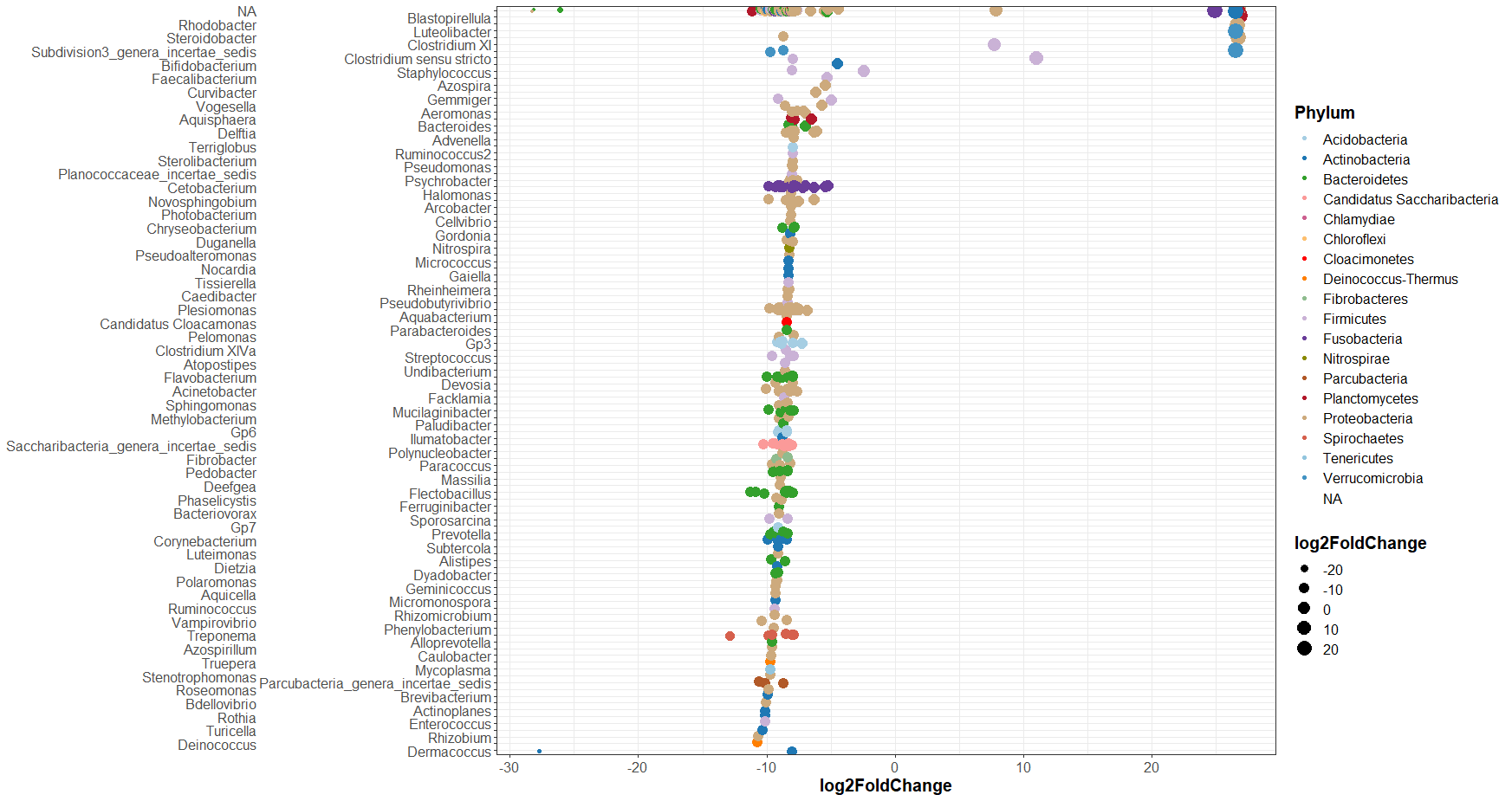


**Supplementary Figure 5.** The significantly abundant ASVs in the anterior intestine of the outbred group compared to the inbred group of tilapia. Size of the dots changes with the fold change.

**
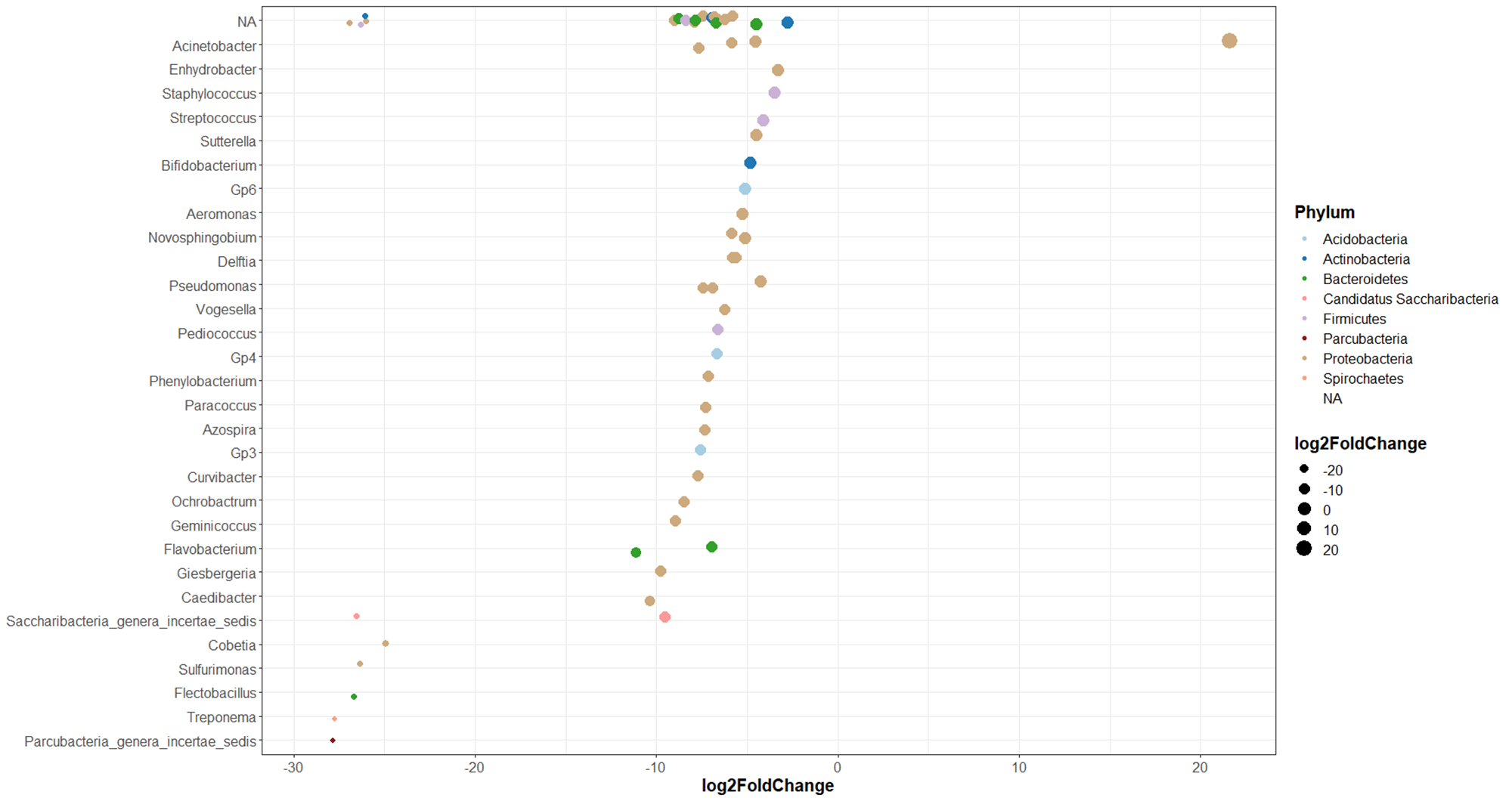
**

**Supplementary Figure 6.** The significantly abundant ASVs in the posterior intestine of the outbred group compared to the inbred groups of tilapia. Size of the dots increases with the fold change.


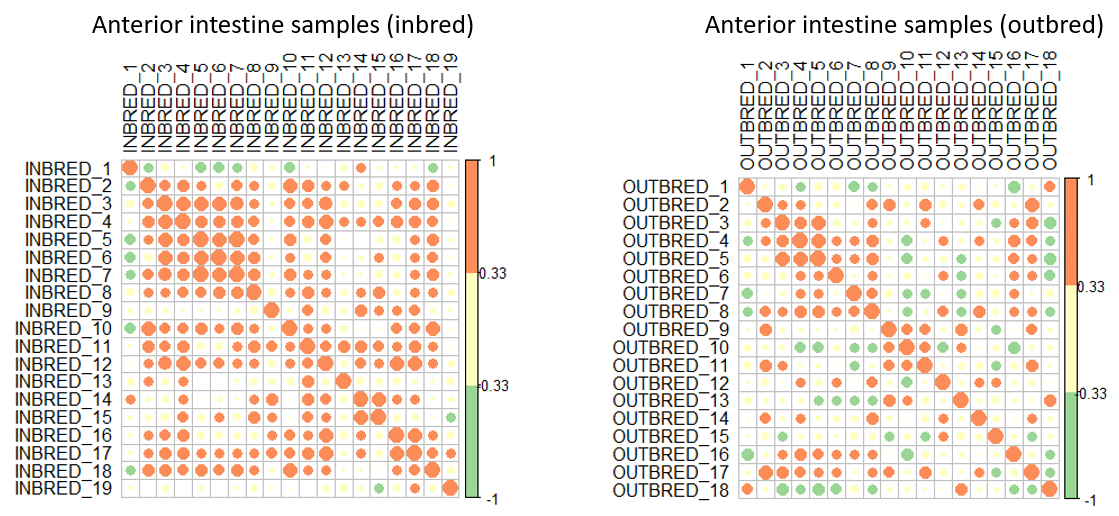


**B**

**A**


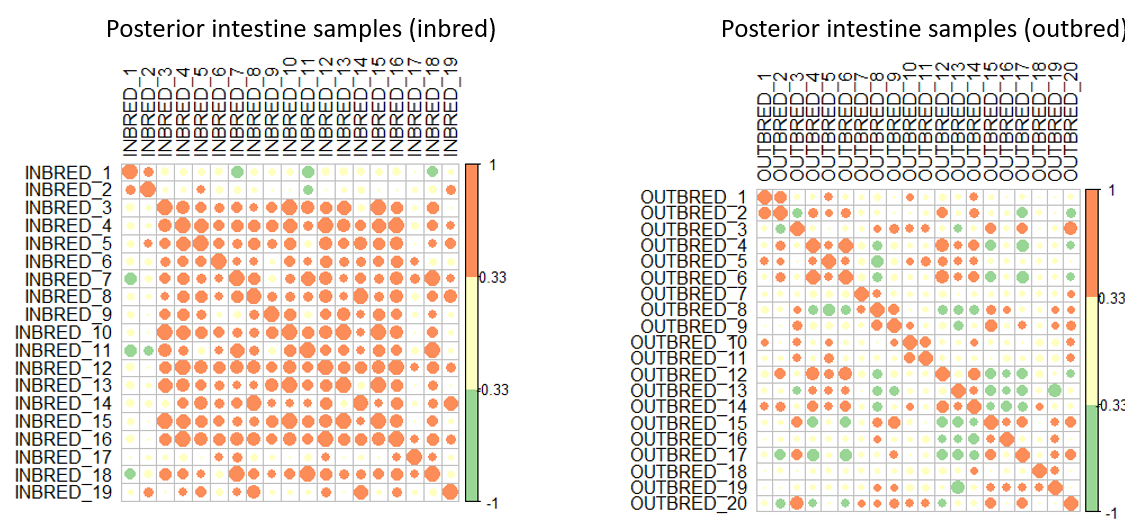


**D**

**C**

**Supplementary Figure 7.** Similarity in the abundance of the core microbiome in the intestine samples of the inbred group. Orange and green circles indicate positive and negative correlation, respectively. Size of the circles shows the degree of association between variables.


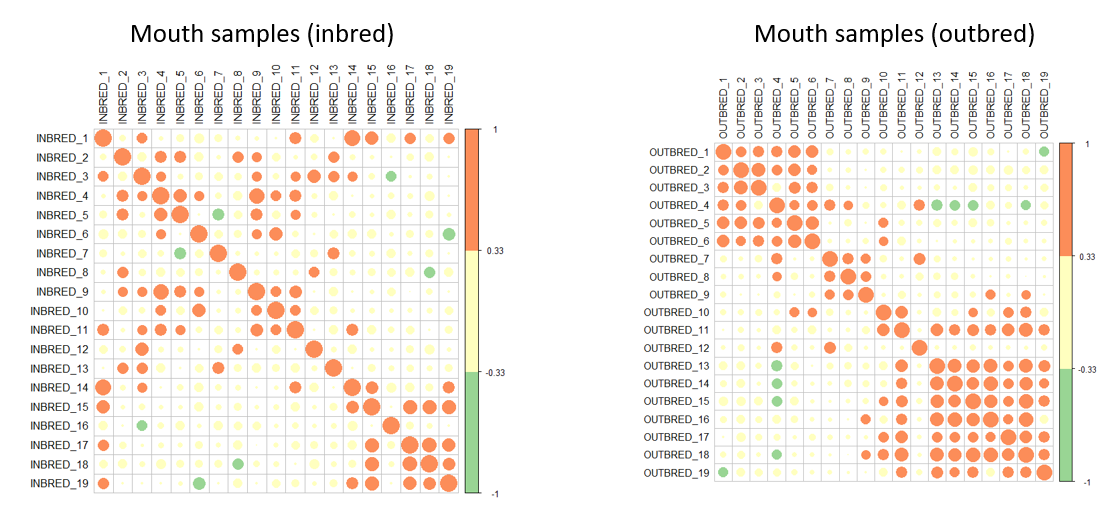


**A**

**B**

**Supplementary Figure 8.** Dissimilarity in the abundance of the core microbiome in the mouth samples of the inbred group. Orange and green circles indicate positive and negative correlation, respectively. Size of the circles shows the degree of association between variables.

**
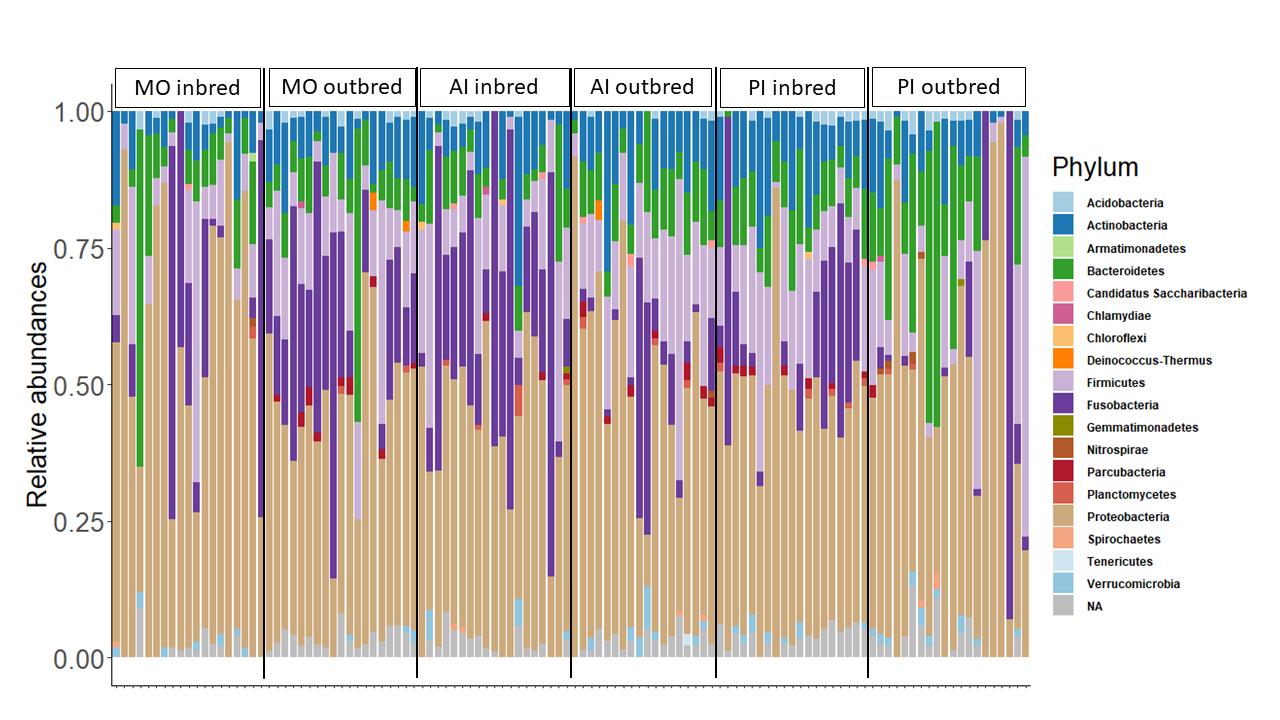
**

**A)**


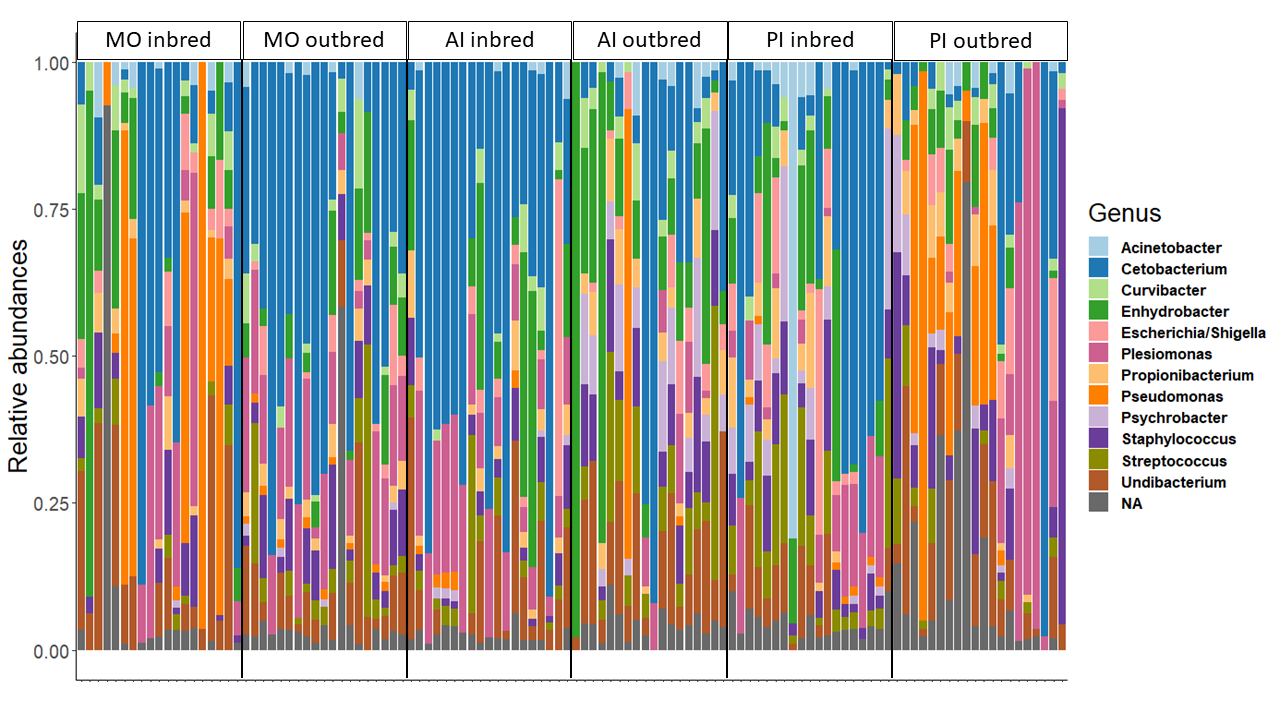


**B)**

**Supplementary Figure 9. A)** Relative abundance of bacteria (phylum-level) in the samples of the inbred and outbred groups of tilapia. **B**) Relative abundance of bacteria at the genus-level.

**Supplementary Table 1.** Proportion of different bacteria in the inbred and outbred groups of tilapia.

| Group | Phyla | Body site | Proportion %* |
| --- | --- | --- | --- |
| Inbred | Proteobacteria | Mouth | 33.07 |
|  | Bacteroidetes |  | 6.79 |
|  | Fusobacteria |  | 2.92 |
|  | Firmicutes |  | 5.16 |
|  | Actinobacteria |  | 3.30 |
|  |  |  |  |
| Outbred | Proteobacteria |  | 25.09 |
|  | Bacteroidetes |  | 9.32 |
|  | Fusobacteria |  | 4.88^•^ |
|  | Firmicutes |  | 4.94^•^ |
|  | Actinobacteria |  | 4.47 |
|  |  |  |  |
| Inbred | Proteobacteria | Anterior intestine | 14.55 |
|  | Bacteroidetes |  | 2.13 |
|  | Fusobacteria |  | 9.77 |
|  | Firmicutes |  | 5.40 |
|  | Actinobacteria |  | 2.84 |
|  |  |  |  |
| Outbred | Proteobacteria |  | 32.85 |
|  | Bacteroidetes |  | 6.59^•^ |
|  | Fusobacteria |  | 6.37^•^ |
|  | Firmicutes |  | 11.90 |
|  | Actinobacteria |  | 7.54 |
|  |  |  |  |
| Inbred | Proteobacteria | Posterior intestine | 21.04 |
|  | Bacteroidetes |  | 2.60 |
|  | Fusobacteria |  | 15.49 |
|  | Firmicutes |  | 7.19 |
|  | Actinobacteria |  | 3.99 |
|  |  |  |  |
| Outbred | Proteobacteria |  | 27.61 |
|  | Bacteroidetes |  | 4.33 |
|  | Fusobacteria |  | 3.89 |
|  | Firmicutes |  | 8.75 |
|  | Actinobacteria |  | 5.06 |
|  |  |  |  |

***Chi-square test detected statistically significant differences between the proportions of the phyla in each breeding group (P** ≤ **0.005).**

**^•^ indicate no statistically significant differences between the phyla in the outbred group.**

**Supplementary Table 2.** Wilcoxon rank-sum test results showing the differences in the alpha diversity of bacteria in the inbred and outbred groups of tilapia.

| Body site | α- diversity | Median | P-value |
| --- | --- | --- | --- |
| Mouth | Species richness | 371 (Inbred) | 0.123 |
|  |  | 473 (Outbred) |  |
|  | Shannon diversity | 36.8 (Inbred)  66.7 (Outbred) | 0.007* |
|  | Simpson diversity | 15.5 (Inbred)  24.5 (Outbred) | 0.078 . |
| Anterior intestine | Species richness | 573 (Inbred) | 0.075 . |
|  |  | 403 (Outbred) |  |
|  | Shannon diversity | 44.7 (Inbred)  72.1 (Outbred) | 0.258 |
|  | Simpson diversity | 17.7 (Inbred)  34.6 (Outbred) | 0.057 . |
| Posterior intestine | Species richness | 514 (Inbred) | 0.113 |
|  |  | 440 (Inbred) |  |
|  | Shannon diversity | 62.3 (Inbred)  120 (Outbred) | 0.224 |
|  | Simpson diversity | 22.1 (Inbred)  54.2 (Outbred) | 0.041 * |

*indicates P < 0.05.
